# Supplementary material for: Phylogenetic factorization of compositional data yields lineage-level associations in microbiome datasets
Source: PeerJ. 2017 Feb 9;5:e2969. doi: 10.7717/peerj.2969 (PMC5345826; doi:10.7717/peerj.2969)
Supplement: Supplemental Information 1 [file peerj-05-2969-s001.pdf]

Supplemental Information: Phylogenetic Factorization of Compositional Data

December 22, 2016

Methods

Zeros

OTU tables are filled with zeros, especially OTU tables from disparate sites. In our tables, ~90% of the entries were zero, and the multiplicative method [3] with a high δ_k would cause taxa present with low sequence counts to have abundances lower than δ_k, and a low δ_k would cause zeros to have an outsized impact on our dataset. For simplicity, we used Aitchison's additive method, setting the remaining zeros to $\delta_k = 0.65$ of a sequence count. Further discussion of how the various methods for dealing with zeros affect phylofactorization is left for future research.

Isometric Log-Ratio Transform

To introduce terminology and notation, we'll summarize the isometric log-ratio (ilr) transform, though see [4] for a more thorough treatment. At its heart, the ilr transform [2] is a change of basis. A traditional compositional vector of D parts, such as the relative abundance of D species in a site, lies in the $D-1$ dimensional simplex, $\mathbf{y} \in \Delta^D$, where y_i represents the coordinates of the corresponding elementary basis, $\{\mathbf{e}_i\}_{i=1}^D$ where

$$\mathbf{e}_{ij} = \begin{cases} 1 & i = j \\ 0 & i \neq j \end{cases}. \tag{1}$$

The ilr transform projects the composition onto a $D-1$ element basis that can be seen as representing sequential binary partitions of the composition [1] (see figure S1). The coordinates of an ILR transform, referred to as "balances", represent the balance of relative abundances on each side of a sequential binary partition. For an arbitrary split separating the compositions in a group, R with

r elements from the compositions in a group, S with s elements, from the rest, the coordinate, which we'll refer to as $x^*_{R/S}$, are

$$x^*_{R/S} = \sqrt{\frac{rs}{r+s}} \log\left(\frac{g(\mathbf{y}_R)}{g(\mathbf{y}_S)}\right) \tag{2}$$

where $g(\mathbf{y}_R)$ is the geometric mean of all $\mathbf{y}_i$ for $i \in R$. This transform can be inverted, allowing one to analyze the coordinates, $\mathbf{x}$, and obtain estimates of relative abundances, $\mathbf{y}$. Changing variables from $\mathbf{y}$ to $\mathbf{x}$ can be represented by Aitchison projection, $< \mathbf{y}, \mathbf{b}_{R/S} >_a$, on a unit-norm balancing element

$$\mathbf{b}_{R/S} = (0, ..., 0, b_r, ..., b_r, b_s, ..., b_s, 0, ..., 0) \tag{3}$$

where $b_r = \sqrt{s/r(r+s)}$ and $b_s = \sqrt{r/s(r+s)}$. In other words,

$$[\mathbf{b}_{R/S}]_j = \begin{cases} \sqrt{\frac{s}{r(r+s)}} & j \in R \\ \sqrt{\frac{r}{s(r+s)}} & j \in S \\ 0 & otherwise \end{cases} . \tag{4}$$

Interpretation and Intuition

To build intuition about the isometric log-ratio transform, it's helpful to see the balances as a ratio of relative abundance on each side of the partition - if the taxa in R are more abundant, this ratio will be positive. If the absolute abundance of all taxa in R increase geometrically by a factor, α_R, while the abundances of taxa in S remain constant, the coordinate $x^*_{R/S}$ will change by the addition of $\left(\sqrt{(rs)/(r+s)}\right) \log(\alpha_R)$. The ILR serves as a measure of contrast between the two taxa - when the taxa on each side of a partition have the same relative abundances, the ILR coordinate corresponding to the partition will be 0.

The geometric mean is a natural way to group taxa into an aggregate relative abundance. Unlike the total relative abundance, $p_R = \sum_{i \in R} y_i$, the geometric mean produces compatible analysis of the original parts and their amalgamations under Aitchison distances. With the geometric mean as a measure of central tendency and amalgamation, the center of an amalgamation is the amalgamation of the centers and distances between centers of amalgamations are monotonically related to the distances between amalgamations of centers. To build more intuition, we note that the ILR is a specific measure of contrast between taxa, that the geometric means and log-ratios are particular to the compositional nature of the data, and that the ILR can be extended easily to random variables that are not compositional: for Gaussian random variables, the natural way to measure contrast would be to look at the difference between the average abundances of taxa on two sides of a partition. A similar phylofactorization can be conducted for any random variables once researchers have an agreeable measure of central tendency and a compatible measure of contrast between two measures of central tendency (all of which are easily identifiable

by looking for the most useful/informative metric space for a given random variable).

More intuition on the ilr-transform can be obtained from re-writing the balance in equation (2) in several different ways:

$$
\begin{aligned}
x^*_{R/S} &= \sqrt{\frac{rs}{r+s}} \log\left(\frac{g(\mathbf{y}_R)}{g(\mathbf{y}_S)}\right) && (5)\\
&= \sqrt{\frac{rs}{r+s}} \left(\overline{\log(\mathbf{y}_R)} - \overline{\log(\mathbf{y}_S)}\right) && (6)\\
&= \sqrt{\frac{rs}{r+s}} \log\left(\left(\prod_{i\in R}\prod_{j\in S}\frac{y_i}{y_j}\right)^{\frac{1}{rs}}\right) && (7)\\
&= \sqrt{\frac{r+s}{rs}} \sum_{i\in R} \log\left(\frac{y_i}{\prod_{j\in(R\cup S)} y_j^{\frac{1}{r+s}}}\right). && (8)
\end{aligned}
$$

The first expression of the balance, equation (5), writes the balance as a log-ratio of geometric means between two groups. The second, equation (6), reveals that the balance is proportional to the difference between the means the log-transformed abundances of the taxa in the two groups. The third, equation (7), expresses the ratio of geometric means in equation (5) as the geometric mean of all possible ratios between taxa in the different groups. Together, (5-7) show that the balances from the ilr transforms indicate how different, on average, the taxa on two sides of a split are. When an ilr coordinate has a strong correlation with an independent variable, it means that the taxa split by that coordinate are different and that difference grows, shrinks, or changes sign with the indendent variable.

The final expression for the ilr coordinate, equation (8), reveals the coordinate to be proportional to an additive amalgamation of the centered-log ratio transformed coordinates, $z_i = \log\left(y_i/g(\mathbf{y})\right)$, of the sub-compositional data from one of the two groups being split. Intuitively, if we had absolute abundance data, one can assess the significance of a clade by amalgamating (adding) the abundances of members in that clade and then performing regression on the amalgamated abundance. As seen in equation (8), the ilr transform follows this intuition, except the abundances to be amalgamated are the clr-abundances within the sub-composition of all taxa split at a given partition. Combining all of these perspectives, the ilr coordinate can be intuited as a measure of contrast between two groups written as either a difference between their mean abundance or an amalgamated clr-abundance of one of the two groups. A significant association of an ilr coordinate can indicate that the ilr coordinate identifies groups with, on average, some meaningful distinction. This is precisely what is desired when searching for phylognetic factors in microbiome data: the edges of the phylogeny which meaningfully distinguish taxa with different functional traits.

The ilr transform for a phylogeny rooted at the most-recent common ancestor

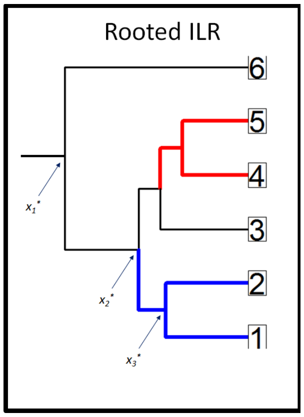

Figure 1: The ilr transform on a rooted phylogeny

is well-suited for analyzing effects isolated within clades but poorly suited for more general effects causing geometric increases in taxa. For example, consider the partition illustrated in figure S1. The third balance, x_3^*, corresponds exactly to the ratio of the relative abundance species 1 to the ratio of relative abundance of species2. Suppose the species are sampled in environments E_1 and E_2 and have respective relative abundances y_{11} and y_{21} in site 1 and abundances y_{12} and y_{22} in site 2. If the absolute abundance of species 1 went up by a factor of α from E_1 to E_2, and the abundance of all other species remained unchanged, the balances of the taxa in two environments, then, would be $x_3^*(E_2) = x_3^*(E_1) + \log(\alpha)$. However, all balances along the path from the node splitting species 1 and 2 to the root of the tree will also see an increase. The bases are orthogonal, but their coordinates are correlated and inference based on these coordinates would not elegantly identify species 1 as the species which changed by a factor of α.

The ilr transform of the rooted phylogeny will likely still find its use, however, in other questions where comparisons of sister taxa are more appropriate. For instance, if a researcher is interested in finding clades with zero-sum competition over a limiting resource contained within the clade, the ilr transform may be well-suited to identify these clades and isolate their internal, zero-sum competition from the rest. In another sense, the ILR transform of the rooted phylogeny compares sister taxa while controlling for their shared evolutionary history. However, for more general purposes of correctly estimating effects α_i for a set of affected taxa, $i \in A$, we use phylofactorization, an approach to constructing an ilr basis which is consistent to the general group-structure of the phylogeny while not rooted at the common ancestor.

PhyloFactor

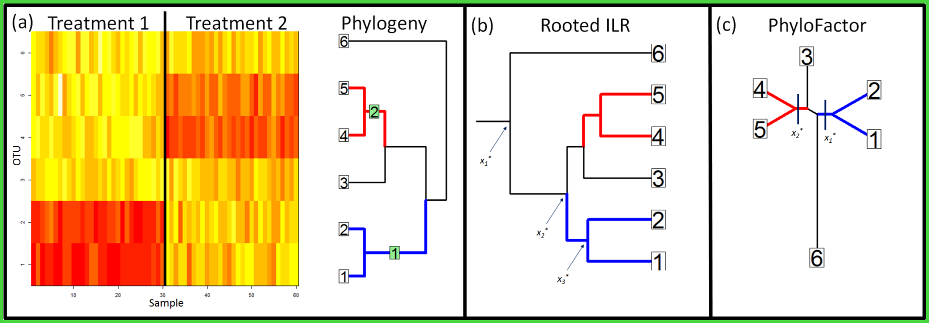

Phylofactorization of Compositional Data

(a) The Challenge: Traits driving community structure or responses to meta-data can be shared within clades, either because they are vertically transmitted or because they can only be horizontally transmitted within a monophyletic group. Here, we present a method to develop an appropriate phylogenetic partition - identifying clades {1,2} and {4,5} in the figure - which yields easily-interpretable inferences on the tree of life and can be a tool for dimensionality reduction by collapsing species into their appropriate clades. Amplicon sequence-count data are inherently compositional - they contain only information about relative and not absolute abundances - and so developing a method for phylogenetic inference of compositional data will allow researchers studying microbiomes to fill in the microbial tree of life with clades with known associations with treatments or other meta-data.

(b) Rooted ILR: The isometric log-ratio transform constructs an orthonormal basis for the sample space of compositional data. The coordinates x_i^*, called "balances", correspond to ratio of relative abundances on each side of a partition in a sequential, binary partition (see [2, 1] for more detailed information). Applying the ILR transform to the rooted phylogeny - here referred to as the "rooted ILR" - can be useful for identifying effects contained within clades, such as zero-sum competition of close relatives or perhaps the substitution of one relative for another across environments, but it can also lead to structured residuals and correlated balances (see figure 1).

(c) PhyloFactor: By implementing a greedy algorithm re-rooting and re-ordering the partitions in an unrooted phylogeny, many of the pitfalls of the rooted ILR can be avoided. In its first iteration, PhyloFactor constructs the ILR basis element formed by placing a root at any edge in the unrooted tree, determines which ILR basis element maximizes the objective function, and regresses out the effect of that element. For all subsequent iterations, the process is repeated - roots are considered along all remaining edges - and the subsequent ILR basis elements are orthogonal to all previous basis elements. The first iteration of PhyloFactor identified the first coordinate as the balance of {1,2} relative to {3,4,5,6} and regressed out the clear signal of {1,2} being hyper-abundant in treatment 1. The second iteration identified the balance of {4,5} relative to {3,6}, along with its effect of an elevated abundance of {4,5} in treatment 2. This process can be repeated until we have a complete set of ILR coordinates or until meeting some stopping criterion, such a threshold weakness of signal in the residuals or a threshold percent of variation in the data is explained.

While most factors corresond to edges in the phylogeny, some downstream factors can have some structural uncertainty, or uncertainty in which edge contains the putative trait. In figure (c) above, a final inference between OTUs {3} and {5} would carry uncertainty about exactly which edge carries the trait that causes differential abundance patterns in the two taxa. The structural uncertainty of factor locations needs to be addressed in future work before phylofactorizations can be used to annotate online databases.

In order to identify affected clades, $i \in A$, that change by some factor $\alpha_i(t)$ with some independent variable, t, a more robust approach is to unroot the phylogeny and form a sequential binary partition via a greedy algorithm. At its most basic, the input for this phylofactorization is a dataset, a phylogeny, a formula for regression, and an objective function. Given an OTUTable, `Data`, a phylogeny, `tree`, and an independent variable, `X`, our function, `PhyloFactor(Data,tree,X)` will perform phylofactorization with a default objective function of minimizing variance in the clr-transformed residuals. Because the total variance in a compositional dataset is constant [4], and is equivalent to the sum of variance the ILR balances for any given sequential binary partition, we reduce the computational cost by maximizing the non-normalized explained variance (the difference between the null deviance and the deviance in the regression on ILR coordinates).

Before going further, we define a few terms for clarity and convenience:

Terms

group : a set of taxa.

partition : a split between elements of a group, signified with "|", as in $\{g_1|g_2\}$ which splits the group $\{g_1, g_2\}$ into two groups, $\{g_1\}$ and $\{g_2\}$.

factor : a particular partition chosen in phylofactorization.

bins : the minimal set of groups not split by a given partition. In the set $\{g_1|g_2, g_3|g_4, g_5, g_6\}$, the bins are $\{g_1\}$, $\{g_2, g_3\}$, and $\{g_4, g_5, g_6\}$.

group complement: the complement of a group, g_i, within its bin. In the above example, the group complement of g_4 is $\{g_5, g_6\}$. One could also label $\{g_5, g_6\}$ the group and g_4 its complement.

Algorithm

Phylofactorization iterates through 6 steps, visualized in figure 3 of the main manuscript.

1. Obtain the set of unique groups and their complements corresponding to each remaining edge in the phylogeny and the bins defined by any previous partitions. In figure S1, the first iteration would obtain unique groups $\{n\}_{n=1}^{6}$, $\{1, 2\}$, $\{3, 4, 5\}$, and $\{1, 2, 3, 4, 5\}$.

2. Project the data onto the set of putative ilr basis elements, $\mathbf{b}_g$, corresponding to the balance each group, g over its compliment.

3. Perform regression on the coordinates from (2)

4. Determine g_{max}, the group which maximizes the objective function. By the symmetry of the log-ratio transform, whether regression is done with based on g_{max} or its complement will not affect the choice of g_{max} for objective functions that unaffected by the sign of the ilr coordinate.

5. Add balancing element $\mathbf{b}_{g_{max}}$ to the basis. In effect, this is re-rooting a sub-tree formed by previous partitions along the edge separating g_{max} from its compliment.

6. Repeat steps (1)-(5) until $D-1$ times, or stop according to some stopping function and define the subsequent basis according to the rooted sub-trees.

With deterministic data, Phylofactorization correctly identifies the effects, $\alpha_i(t)$, shared in common by all taxa in each of set of clades $i \in A$, provided that the number of affected clades, $m \leq D-1$ and rest of the clades are unaffected, i.e. $\alpha_j(t) = 1\ \forall t$ and $\forall j \notin A$. If the number of affected clades $m > D-1$, each taxon might have its own effect and phylofactorization, although unable to identify all of the edges causing differential effects, can still partition the tree into edges driving variation in our data. Thus, phylofactorization as described here is a recommended method when researchers anticipate there are a small number of clades with meaningful effects in an experiment, and should be used with caution when the number of traits driving variation in the response variable is larger than the number of taxa.

The choice of objective function is central to the efficacy of phylofactorization and should match the purposes of the researcher. If a researcher is interested in describing the factors driving variation in a community's response to a treatment, the inverse of the residual variance may be an appropriate objective function. If a researcher is interested in identifying which taxa are responsible for shifts in some diversity index in response to treatment, a loss function such as the variance in diversity index between treatments would be suitable. If a researcher exposes a community to a treatment, such as antibiotics, and is interested in the individual clades with the strongest effect to the treatment, objective functions such as first selecting taxa with significant coefficients for regression and then, of those taxa, choose the taxon with the largest response. For all analyses in this paper, our objective function chose the ilr basis element which minimized the residual variance in the clr-transform of the residual compositional dataset. We leave the discussion of appropriate objective functions for phylofactorization to future research.

Output and Interpretation

There are many ways to view the output of phylofactorization. We provide a few examples.

1. **Factor-based analysis:** The output of phylofactorization is a set of factors. Each factor represents an edge in the phylogeny splitting two complementary groups within an bin defined by the previous, incomplete partition. Analysis of factors allows researchers to explore, sequentially, the major clades driving variation in their data. Below are some examples of the questions one could ask of these factors:

   (a) Did the factor split a tip from a complementary group, or did it

identify a clade? The R function phylofactor outputs labels indicating whether one the factor split clades, a tip, or two tips.

(b) If the factor split two groups of taxa, is one of the groups monophyletic? If one group is monophyletic, parsimony assumptions can be introduced to infer that a trait driving an inferred effect is shared in common with the monophyletic group. We leave the application of parsimony assumptions to the researcher, but, for a given factor, the function `phylofactor.summary` includes output on which group(s) in the factor are monophyletic in the full tree being considered. For example, consider the tree depicted in figure S1. If the first factor split OTU 4 from the rest, it would be splitting a tip from a clade, and the tip is monophyletic, while the clade is not. If the second factor split $\{3, 5\}$ from the rest, it would split two clades, $\{3, 5\}$ from $\{1, 2, 6\}$, neither of which are monophyletic. If, instead of 4, the first factor split $\{1, 2\}$ from the rest, it will have split two clades, one of which is monophyletic. If the second factor then split $\{3, 4, 5\}$ from $\{6\}$, it will have split two clades, both of which are monophyletic. inputting the `phylofactor.summary` object into `pf.tidy` yields a concise symmary of the taxa split at the summarized factor, as well as the observed and predicted ratio of geometric means of taxa on each side of the partition.

2. **Bin-based analysis:** At a given level of phylofactorization, there are a set of bins which remain unsplit, and the regression on all upstream factors can be combined to give estimates of the abundances and effect sizes between bins. At a given level of factorization, binning taxa will yield something like an OTU - what we call a "binned" phylogenetic unit or BPU. The organisms in BPUs are not necessarily monophyletic, but rather all taxa within the bins at a given level of factorization are assumed to have the same relative abundances across treatments, and when the predicted abundances of the bin change, the relative abundances all taxa within the bin are assumed to change by the same amount. While analysis of factors allows researchers to explore the sequence of partitions, the analysis of bins allows researchers to examine the groups at a given level of factorization and their effects in different treatements or biotic/abiotic conditions. A few questions that can be analyzed with bin-level analysis:

   (a) Which groups are most affected by a treatment? While factors allow a sequential examination of effects, the effects of nested clades in previous and subsequent factors are not accounted for and could reverse the inferences for a clades. An effect on a final bin from an earlier factor indicating an increase in relative abundance in response to treatment can be reversed by an opposing effect in a later factor, and so statements about affected groups must ultimately be made about the bins. Hence, in our power analysis, we determined the

percent of success for a phylofactorization based on the percent of bins in phylofactorization matching the affected clades.

(b) Cross-validation of the effects of bins. Confronted with a big dataset, a researcher may be interested in factoring a small dataset and then projecting the larger dataset onto a lower dimensional space defined by the factors. We caution that the application of an ilr transform from one factorization to an independent dataset may have the same problems of correlated coordinates and, when removing regressed effects, residual structure. However, there may be techniques for cross-validating the effects on bins which allow researchers to identify clades of interest in big datasets.

(c) Is the size-distribution of groups any different than expected by chance? For a given number of factors, one could simulate the size distribution of bins and ask questions about whether the size distribution of bins in the dataset differs from the size distribution in simulated random partitions. This can allow researchers to determine how basal are the affected edges in the tree of life. A cottage industry of similar questions can be analyzed in combination with factors - what's the probability that the first n factors have the observed ratio of clades to tips if the null hypothesis of randomly drawn partitions were true?

These proposed types of explorations of the data are not an exhaustive list of what can be done with phylofactorization. We recognize that this method is in its infancy, and are excited to see its use be expanded to new choices of objective functions, stopping functions, new greedy algorithms for various deterministic or stochastic decisions and new methods for analyzing factors and bins.

Error Types

We anticipate there being four main types of errors. We can't examine all of them in this already-lengthy manuscript, but we list some fo them here to invite future research:

1. **Standard Type 1 and Type 2 errors:** Each iteration of phylofactorization performs regression on a large number of candidate dependent variables (the ILR-coordiantes corresponding to an edge). Ultimately, we choose the ILR coordinate that maximizes some objective function, and that chosen edge may be false-positives or other edges we haven't chosen false-negatives.

2. **Multiple Hypothesis Testing:** We obtain a sequence of factors, each with P-values obtained from F-tests based on the underlying regression of the ILR coordinate. The P-values, however, may not be analyzed with the standard false-discovery rate tools or Bonferonni corrections, because the P-values are drawn from the *best* candidate ILR coordinate at each stage.

3. **Propagation of error:** With a Type 1 or Type 2 error possible at any iteration of the phylofactorization algorithm, the algorithm may make an error and proceed and the previous errors may propagate to further errors in future iterations. This may be controlled by running many repetitions of a stochastic sampling algorithm instead of the deterministic greedy algorithm proposed here.

4. **Uncertain location of edges**: In special cases where two edges separated by one edge are both chosen as factors (as illustrated in the figure of box S1, sub-plot (c)), a subsequent factor crossing thier connecting edge (e.g. separating OTUs 3 and 6 in Box S1 above) will lead to uncertain assignment of the edge along which a putative trait likely exists (the edge could be the tip to either OTU 3 or 6, or it could be the basal edge connecting $\{3, 4, 5\}$ to $\{1, 2, 6\}$). The stochastic sampling algorithm mentioned above may resolve this, and edges can be assigned a probability of containing a trait based on the number of simulations containing a particular edge as a factor. Alternatively, annotations to an online tree can apply to the chain of edges over which there is uncertainty, perhaps weighting each branch's probability of being the site of a mutation by its relative length in the chain.

Relation to Factor Analysis

There is an art to defining new terms in science, and we are not artists but have nonetheless deliberately included the word "factor" in the new term, "phylofactor", in an effort to connect our method to factor analysis and matrix factorization. To avoid confusion, we want to clarify what we mean by a "factor" in phylo-"factorization", and be explicit about how our method relates to factor analysis.

For a real-valued vector of D dependent variables in sample $j \in \{1, ..., n\}$, $\mathbf{z}_j \in \mathbb{R}^D$, with a mean across samples $\mu \in \mathbb{R}^D$, factor analysis aims to find a set of $K \leq D$ unobserved latent variables or "factors", $\mathbf{F}_k \in \mathbb{R}^n$, and a set of K loadings $\mathbf{L}_k \in \mathbb{R}^D$ to minimize the residual varaince, ϵ^2. Defining the standardized observation matrix $[\mathbf{Z}]_{\cdot,j} = \mathbf{z}_j - \mu$ factor analysis aims to find $\mathbf{F}$ and $\mathbf{L}$

$$\mathbf{Z} = \mathbf{L}\mathbf{F} + \epsilon \tag{9}$$

which minimize the off-diagonal elements of the noise covariance matrix, $\epsilon\epsilon^T$. "Factor" analysis is a type of matrix "factorization" because when $K = D$ we can completely factor our data matrix into the product of two matrices, $\mathbf{L}$ and $\mathbf{F}$.

Phylo-"factorization", in its most general form, is the process of sequentially choosing ILR basis elements that correspond to structures edges in the phylogeny and maximize some objective function. In the context of equation (S9) above, the centered log-ratio transformed data are $\mathbf{Z}$ and the ILR basis elements from equation (S3) are the loadings $\mathbf{L}_k$. For a direct link between phylofactor and factor analysis, and for phylofactorization to be a matrix factorization, the

factors are the projections of CLR data onto the loadings / balancing element whose balances maximized the objective function (i.e. the "factors" are the observed balances, x^*). In this sense, phylofactorization is a change of basis into coordinates which correspond to "interesting" edges in the phylogeny, where "interesting" is defined by the objective function.

Throughout the paper, we refer to "edges maximizing the objective function" as "factors", e.g. saying that a "factor" splits a bin in two or a "factor" corresponding to an edge. Our use of "factor" here, which may seem to be more accurately described as the "loading" that strictly defines the bin-separation and correspond to the edge, is intended to be consistent with the other feature of factors in factor analysis - that they are latent variables. The reason for using the evolutionary tree as a scaffolding to constrain the set of possible loadings is to allow inference about latent or unobserved biological features - a trait, perhaps - which could account for differential abundance patterns across an edge in the tree. Thus, there would be a "factor" - a latent variable - corresponding to the edge, separating the bin of amniotes into bins of birds and other amniotes, which can later be observed as "feathers", "wings". and other traits unique to birds.

Thus, phylofactorization chooses loadings and can be interpreted as a constrained factor analysis whose loadings correspond to balancing elements constructed from edges in the phylogeny and whose factors are the balances corresponding to those balancing elements. When a researcher says "we identified a phylogenetic factor predicting inflammatory bowel disease in a patient", such a statement should be interpreted as: "We have found an edge in the phylogeny that can be used to predict the presence of inflammatory bowel disease by constructing an ILR balancing element partitioning the taxa on each side of that edge," and one can immediately hypothesize that a latent, vertically transmitted trait(s) explain these predictive, differential abundances of taxa on each side of the edge.

Computational Benchmarking

The computational costs of phylofactorization for large datasets can be large, but the multiple generalized linear models on each of the candidate ilr-coordinates can be parallelized to allow the computationally-intensive phylofactorization to be performed on servers and clouds. To get a sense of the computational costs of phylofactorization, we provide some benchmarks by simulating the phylofactorization of random datasets. We looked at all combinations of `D`$\in \{30, 96, 300, 948, 3000\}$, `p`$\in \{10, 32, 100, 316, 1000\}$, `nfactors`$\in \{1, 2, 3, 4\}$, and `ncores`$\in \{1, 2, 4\}$ and ran simulations on a home desktop with an 3.30 GHz AMD FX-6100 six-core processor.

The benchmarks are shown below in figure S2. The current algorithm for phylofactorization in the R package `phylofactor` performs 1-clade factorization for `D`$= 3,000$ and `p`$= 1,000$ in ~1,000 seconds on a home desktop without

employing parallelization. The phylofactorization of a dataset is highly parallelizable because most of the work is in amalgamating the data for many groups and computing regressions of many ilr-coordinates against independent variables. The function `PhyloFactor()` accepts an argument `ncores` which allows user-friendly parallelization of phylofactorization. The speedup from parallelization can be significant, especially for big datasets as the percent of the code that is parallelizable approaches 1 for large numbers of OTUs, `D`.

References

[1] Juan José Egozcue and Vera Pawlowsky-Glahn. Groups of parts and their balances in compositional data analysis. *Mathematical Geology*, 37(7):795–828, 2005.

[2] Juan José Egozcue, Vera Pawlowsky-Glahn, Glòria Mateu-Figueras, and Carles Barcelo-Vidal. Isometric logratio transformations for compositional data analysis. *Mathematical Geology*, 35(3):279–300, 2003.

[3] Josep A Martín-Fernández, Carles Barceló-Vidal, and Vera Pawlowsky-Glahn. Dealing with zeros and missing values in compositional data sets using nonparametric imputation. *Mathematical Geology*, 35(3):253–278, 2003.

[4] Vera Pawlowsky-Glahn and Antonella Buccianti. *Compositional data analysis: Theory and applications*. John Wiley & Sons, 2011.

Supplemental Figures

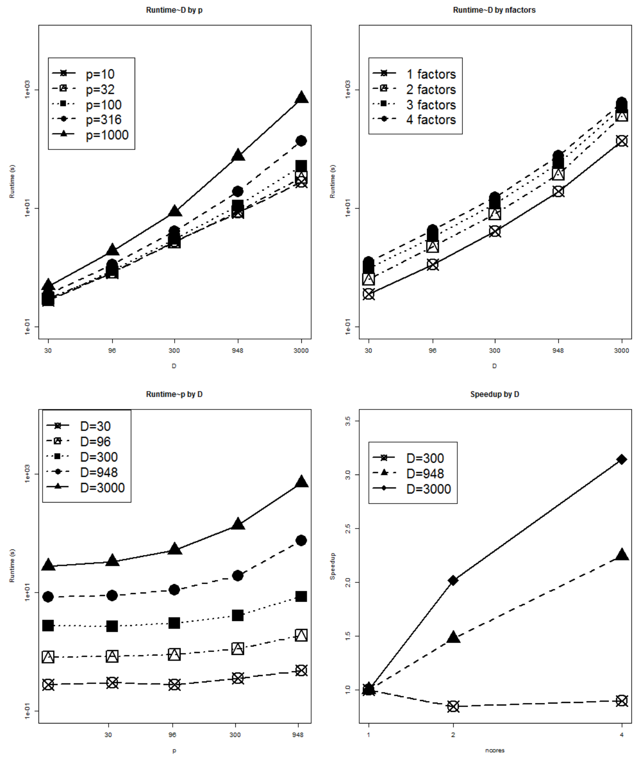

Figure 2: Runtime (in seconds) and as a function of the number of species, `D`, the number of samples, `p`, and the number of factors, and the speed-up as a function of the number of cores for `choice='var'` (the objective function of minimizing residual variance in the entire dataset). The simulation time for the current algorithm appears to increase hyper-exponentially in `D` and `p`, however, with increasing `D` and `p` the speedup from parallelization increases and the precent of the code which is parallelizable gets close to 1 for large `n` and `p`. The current function, `PhyloFactor`, accepts the argument `ncores` for efficient parallelization of phylofactorization on servers, especially for large datasets. Unless noted otherwise, the default parameter values used for plotting are `D=316, p=316, ncores=1, nfactors=1`, and `choice='var'`.

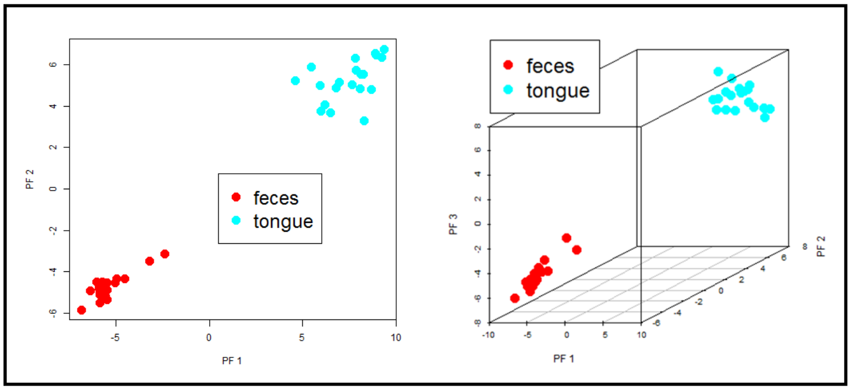

Figure 3: Phylofactorization produces orthogonal balancing elements which can be used for ordination-visualization and dimensionality reduction. Here, the "PF", or phylofactors, represent ratios between clades. PF1, is a re-scaled log-ratio of the Actinobacteria and many Proteobacteria against all the rest (see figure S5). PF2 is a re-scaled log-ratio of Bacilli (see figure S6) from the paraphyletic remainder of the first factor. PF3 is the ratio of Prevotella against the paraphyletic remaining OTUs from the first & second factors. Ordination-visualization performed by phylofactorization lends itself to a very useful, biological interpretation: the three variables capture edges in the phylogenetic tree along which putative traits may have arisen, traits which differentiate organism's responses to sample sites, environmental gradients or treatments.

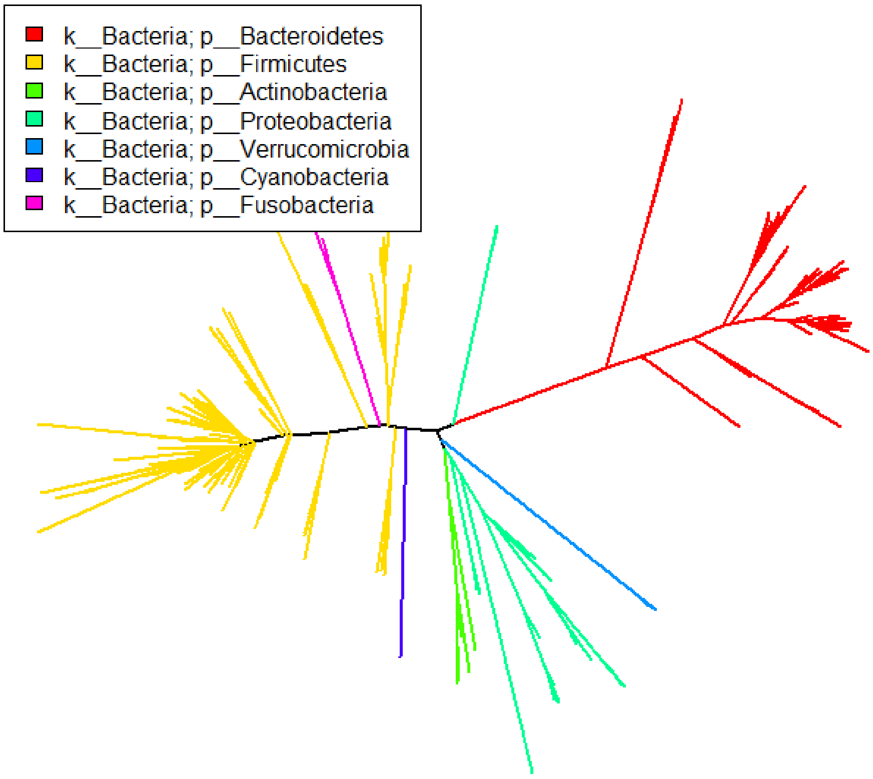

Figure 4: Phylogeny of dataset color-coded by phylum and color-coded phylogeny of the first factor.

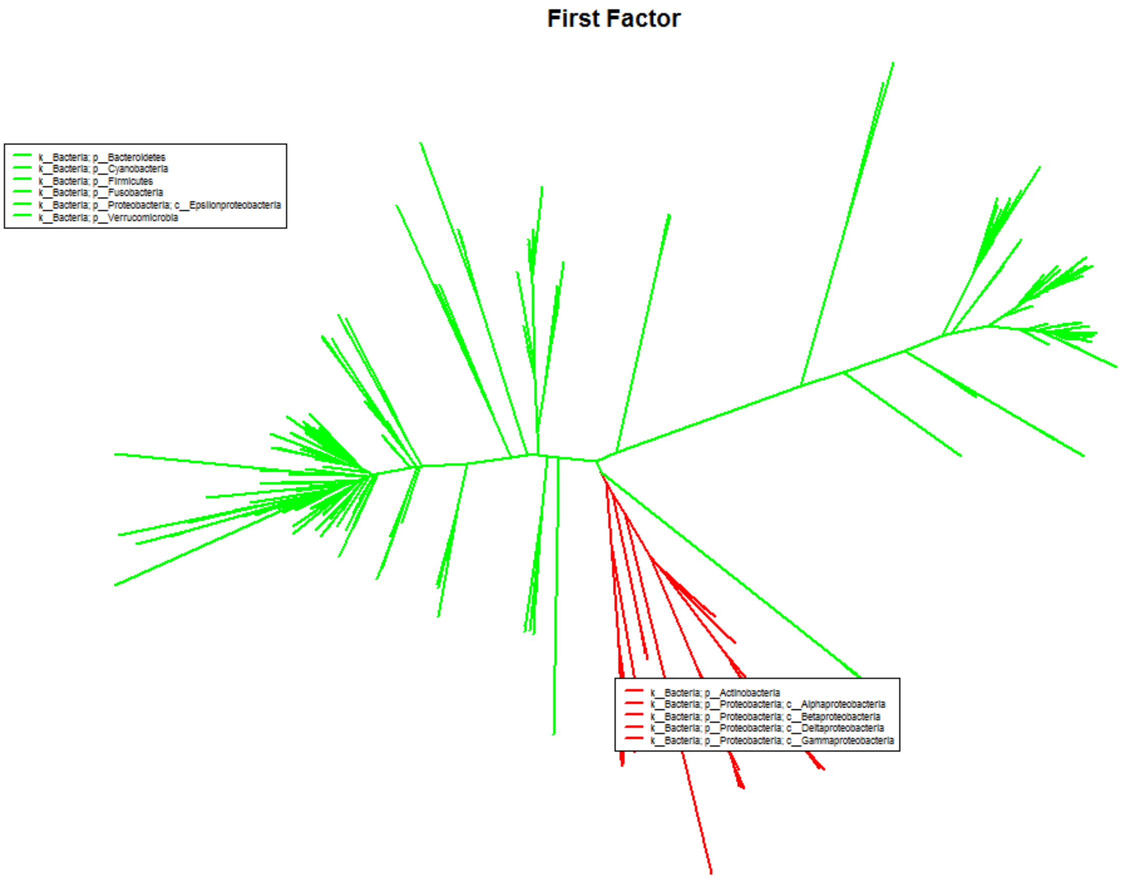

Figure 5: The first factor ($P = 4.90 \times 10^{-30}$) split Actinobacteria and Alpha-, Beta-, Gamma-, and Delta-proteobacteria from Epsilonoproteobacteria and the rest of the tree, with the Actinobacteria and non-Epsilono-proteobacteria being 0.4x as abundant as the rest in the gut and 3.7x as abundant as the rest in the tongue (figure S4). The Actinobacteria identified as being more abundant in the tongue include four members of the plaque-associated family Actinomyceteae, one unclassified species of the mucosa-associated genus *Cornybacterium,* three members of the mouth-associated genus *Rothia,* and one unclassified species of the vaginal-associated genus *Atopobium.* The Alpha-, Beta-, Gamma- and Deltapmroteobacteria grouped with the Actinobacteria consisted of 31 OTUs, including the genera Haemophilus, Cardiobacterium, Neisseria, Lautropia, organisms known to live in the oral-pharyngeal region. The Epsilonoproteobacteira split from the rest were three unclassified species of the genus *Campylobacter*, a genus well known to colonize the small and large intestines.

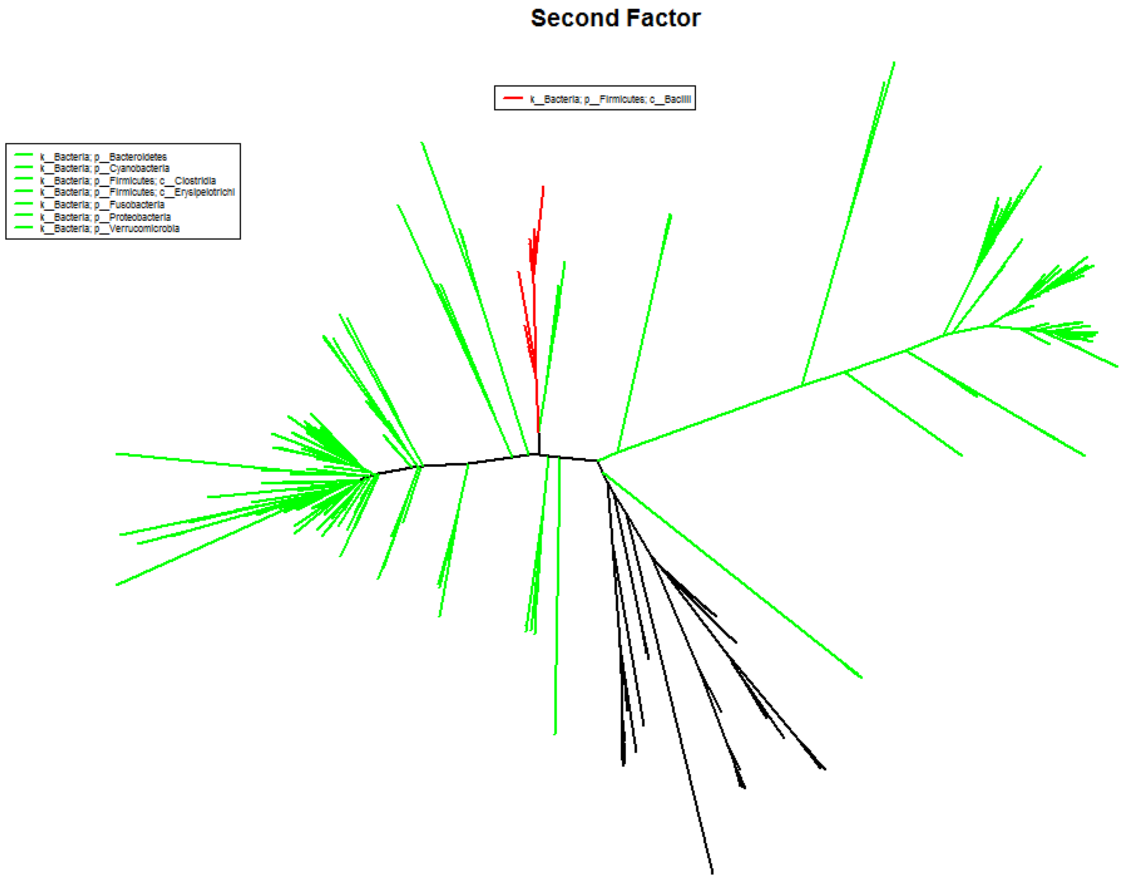

Figure 6: The second factor ($P = 1.15 \times 10^{-31}$) splits 16 Firmicutes of the class Bacilli from the obligately anaerobic Firmicutes class Clostridia and the remaining paraphyletic group containing Epsilonproteobacteria and the rest as described in the first factor. The Bacilli are, on average, 0.3x as abundant in the gut as the paraphyletic remaining OTUs and 3.9x as abundant in the tongue. The 16 Bacilli OTUs factored here contain 12 unclassified species of the genus *Streptococcus,* well known for its association with the mouth, one member of the genus *Lactococcus*, one unclassified species of the mucosal-associated genus *Gemella*, and two mebmers the family Carnobacteriaceae often associated with fish and meat products.

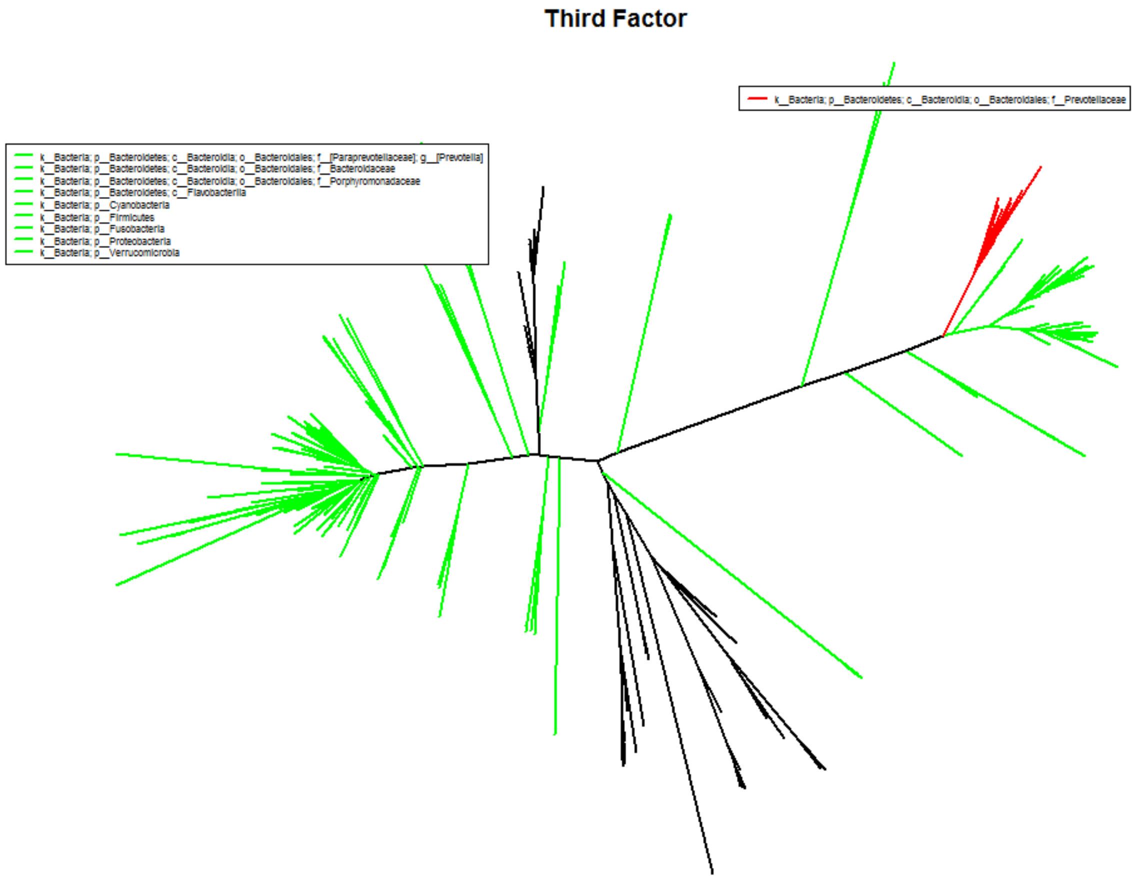

Figure 7: The third factor ($P = 1.37 \times 10^{-28}$) separated 15 members of the Bacteroidetes family Prevotellaceae from all other Bacteroidetes and the remaining paraphyletic group of OTUs not split by previous factors. The Prevotellaceae split in the third factor were all of the genus *Prevotella,* and found to have abundances 0.3x as abundant in the gut and 4.0x as abundant in the tongue as the other taxa from which they were split.

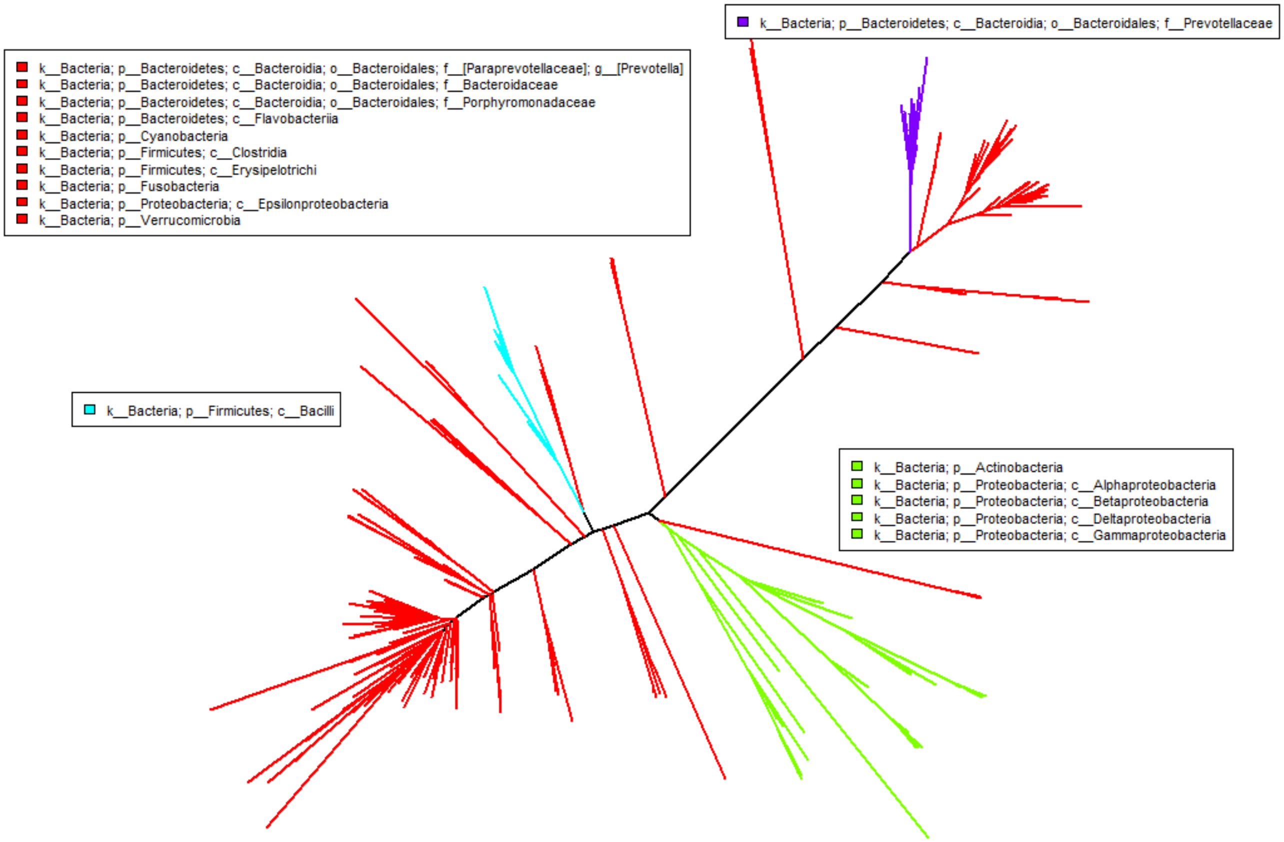

Figure 8: The first three factors from phylofactorization partition the global phylogeny into four "bins" or un-split groups of taxa. Phylofactorization can be seen as a dimensionality reducing technique that reduces our dataset of 290 OTUs into a dataset of 4 "binned" phylogenetic units (what we'll call BPUs). Successive phylogenetic factors can have competing effects - for example, while amniotes are more likely to live on land and fresh-water due to a basal evolutionary event (leading to eggs preventing desiccation, an adaptation to life on land), more distal lineages (e.g. Cetaceans, Pinnipeds, marine turtles and sea snakes) subsequently evolved to return to the oceans. Factor-based analysis tells us the location of putative traits and evolutionary transitions, and bin-based analysis - grouping the un-split taxa and performing regression on these BPUs - allows the prediction abundances and functional ecology conditioned on the set of traits deemed to be important from phylofactorization.
