# Supplementary figures and images for: Phylogenetic factorization of compositional data yields lineage-level associations in microbiome datasets
Source: PeerJ. 2017 Feb 9;5:e2969. doi: 10.7717/peerj.2969 (PMC5345826; doi:10.7717/peerj.2969)

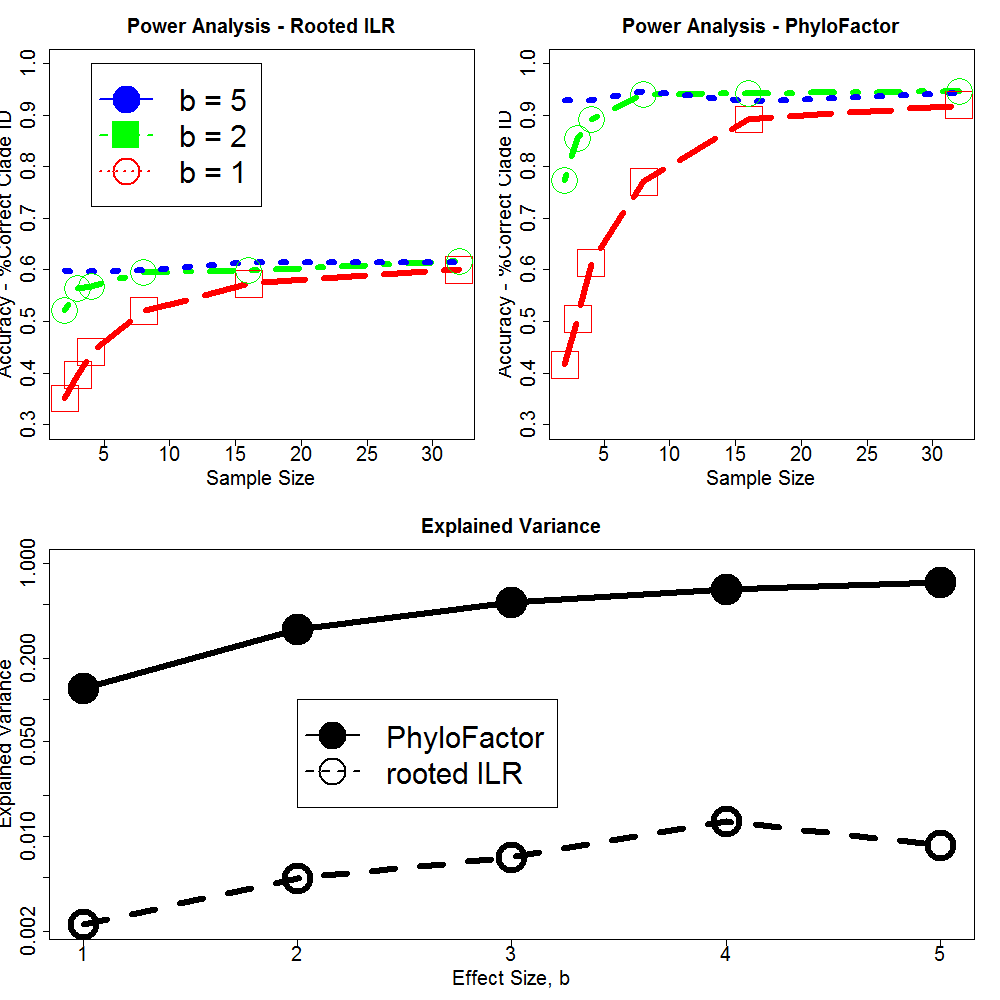

Supplement: Supplemental Information 2 [file peerj-05-2969-s002.zip › PhyloFactor Power Analysis/3_Clade_power_analysis_and_ExVar.tiff]

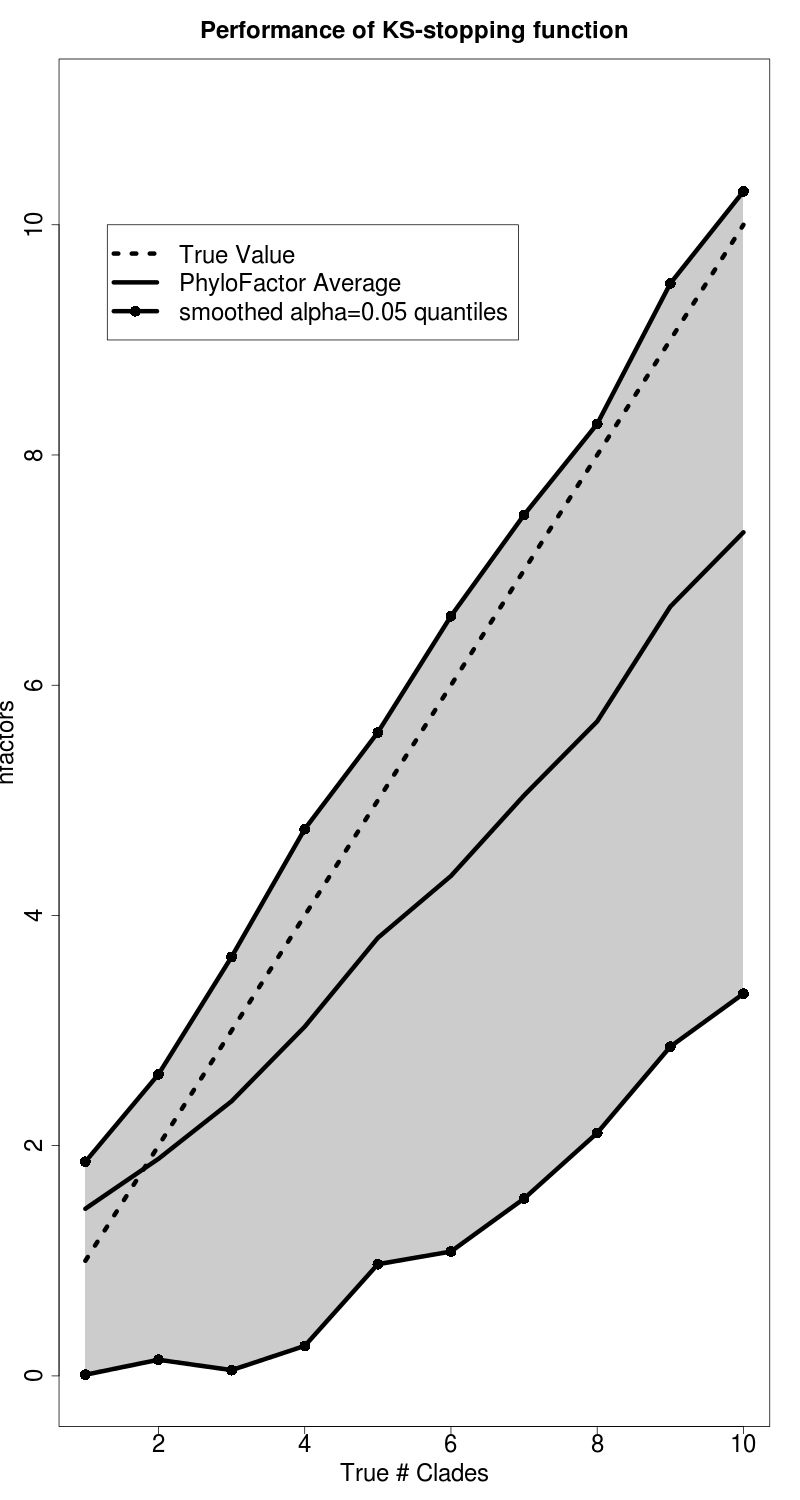

Supplement: Supplemental Information 2 [file peerj-05-2969-s002.zip › PhyloFactor Power Analysis/Nfactors_Stop_Fcn_Checking.tiff]

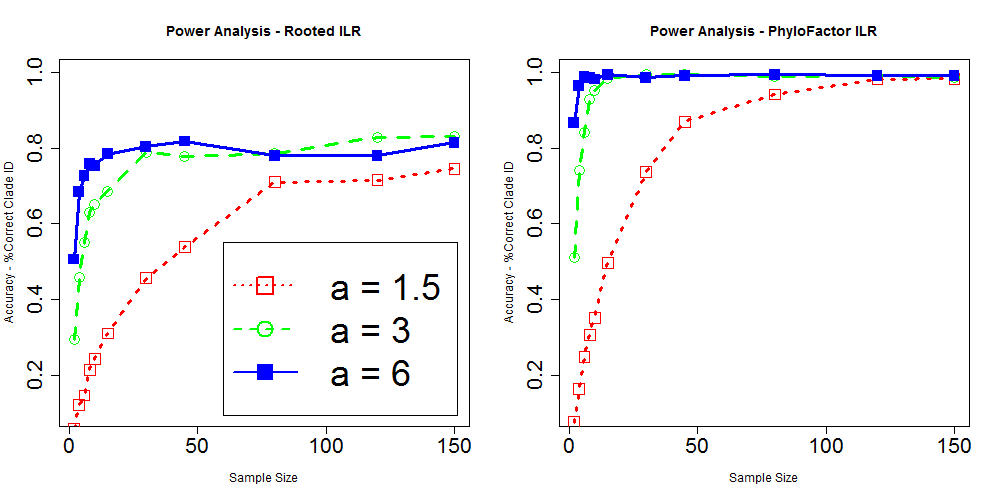

Supplement: Supplemental Information 2 [file peerj-05-2969-s002.zip › PhyloFactor Power Analysis/Power-Analysis_1_clade.tiff]
