# Supplementary material for: Phylogenetic factorization of compositional data yields lineage-level associations in microbiome datasets
Source: PeerJ. 2017 Feb 9;5:e2969. doi: 10.7717/peerj.2969 (PMC5345826; doi:10.7717/peerj.2969)
Supplement: Supplemental Information 5 — How to use, visualize and customize phylofactorizations with the R package ‘phylofactor’. [file peerj-05-2969-s005.html]

phylofactor Tutorial


# phylofactor Tutorial

#### *by Alex Wasburne*

Welcome to phylofactor!

In this tutorial, we’ll download the R package `phylofactor`, and then perform, summarize & visualize phylofactorization of a real microbiome dataset. Slowly, we’ll introduce some more advanced functionality before diving head-first into the guts of the package and the full generality of phylofactorization.

# Downloading

First, let’s download the package:

```
devtools::install_github('reptalex/phylofactor')
```

If you had any trouble downloading the package, the most common errors are missing dependencies. If this happens to you, try to read the errors, find out which packages were not found or failed to load, and then install them individually. For example, if the “package.name” package was not found or failed to load, try: `install.packages('package.name')`

# Getting Started

The workhorse of the phylofactor package is the function `PhyloFactor`. It contains a useful “Help” file with examples of how to use its more advanced functionality, including some not covered in this tutorial.

```
library(phylofactor)
? PhyloFactor
```

The package contains a dataset used in our paper, “FTmicrobiome”. FTmicrobiome contains sequence counts of bacteria from fecal and tongue body sites collected from two humans over time (Caporaso et al. 2011 - an awesome paper whose open access made this method possible). From the time series, we randomly sampled 10 timepoints from each of the two people for each of the two body sites, yielding 40 samples in all, 20 from the feces and 20 from the tongue.

Let’s load the data:

```
data("FTmicrobiome")
```

This dataset is a list containing the necessary ingredients for PhyloFactor:

## Ingredients:

### 1) OTUTable

```
OTUTable <- FTmicrobiome$OTUTable   #Our OTU table. Rows are OTUs and columns are samples
OTUTable[1:5,1:5]
```

```
##         feces feces feces feces feces
## 359105      2     0     0     8     5
## 4371046     0     0     0     0     0
## 191468      0     0     0     0     0
## 362389      0     0     0     0     0
## 292255      0     0     0     0     0
```

### 2) Independent variable(s)

```
body.site <- FTmicrobiome$X         #Our independent variable
str(body.site)
```

```
##  Factor w/ 2 levels "feces","tongue": 1 1 1 1 1 1 1 1 1 1 ...
```

### 3) Phylogenetic tree

```
tree <- FTmicrobiome$tree           #Our phylogeny
tree
```

```
## 
## Phylogenetic tree with 2713 tips and 2711 internal nodes.
## 
## Tip labels:
##  558531, 565292, 345753, 551295, 842714, 327887, ...
## Node labels:
##  , 0.868, 0.934, 0.982, 0.475, 0.859, ...
## 
## Unrooted; includes branch lengths.
```

The tips ### 4) Taxonomy

```
taxonomy <- FTmicrobiome$taxonomy   #Our taxonomy
taxonomy[1:3,]
```

```
##       OTU_ID
## 55158 558531
## 25035 565292
## 9257  345753
##                                                                                        taxonomy
## 55158 k__Bacteria; p__Firmicutes; c__Clostridia; o__Clostridiales; f__Lachnospiraceae; g__; s__
## 25035 k__Bacteria; p__Firmicutes; c__Clostridia; o__Clostridiales; f__Lachnospiraceae; g__; s__
## 9257  k__Bacteria; p__Firmicutes; c__Clostridia; o__Clostridiales; f__Lachnospiraceae; g__; s__
```

The taxonomy is a data frame with two columns. The first column contains the OTU IDs as found in the phylogenetic tree. The second column contains the taxonomy corresponding to each OTU. The only rule for the format of the taxonomy is that the levels must be separated by “;”.

## Filtering - Focusing on ‘common’ taxa (not necessary)

Let’s focus our efforts on common OTUs. The choice of OTU filtering depends on the interests of the researcher - some research questions will want to pay much more attention to rare taxa. Be deliberate about filtering (and treatment of zeros) to best answer your unique question.

In this case, we’re interested in the phylogenetic factors of commonly found taxa, where “common” taxa are defined (subjectively) as those found in over 25% of the samples (>10 samples).

```
common.otus <- which(rowSums(OTUTable>0)>10)

OTUTable <- OTUTable[common.otus,]                        
tree <- ape::drop.tip(tree,setdiff(tree$tip.label,rownames(OTUTable)))  #trim our tree accordingly
```

# PhyloFactorization

We’re all set to phylofactor.

The default phylofactorization is done through a regression model `Data~X` where `Data` is the ILR coordinate and `X` the independent variable. `PhyloFactor` will choose the edge whose regression model maximizes the difference between the null deviance and residual deviance. Intuitively, we’re picking the edge for which we can “explain the most variance” in the data through regression.

We’ll get into more details later. For now, let’s keep it simple and phylofactor these data.

Let’s look at the first three factors by setting `nfactors=3`:

```
PF <- PhyloFactor(OTUTable,tree,body.site,nfactors=3)
class(PF)
```

```
## [1] "phylofactor"
```

The output of `PhyloFactor` is a “phylofactor” object. This object contains many features:

```
names(PF)
```

```
##  [1] "Data"                "X"                   "tree"               
##  [4] "glms"                "terminated"          "groups"             
##  [7] "factors"             "basis"               "ExplainedVar"       
## [10] "nfactors"            "bins"                "bin.sizes"          
## [13] "Monophyletic.clades"
```

1. “Data”: the compositional data matrix used for phylofactorization. This matrix may differ from the input OTU table, as `PhyloFactor` trims rows that are not in the tree, replaces zeros with 0.65, and converts these sequence counts to relative abundances. If you have a preferred method of dealing with zeros it’s necessary to do that before inputting the data into `PhyloFactor`.
2. “X”: the independent variable or data frame of independent variables.
3. “tree”: the unrooted phylogeny whose tips are the rownames of “Data”.
4. “glms”: For the default regression-phylofactorization, `PhyloFactor` outputs the `"glm"` objects from the factored edges.
5. “terminated”: logical, indicating whether or not the iterations were terminated due to stopping criteria.
6. “groups” : A list of length “nfactors” whose elements are two-element lists. Each two-element list contains the row numbers of the OTUs being split at each factor (“Group1” and “Group2” for each factor).
7. “factors”: A convenient summary tool, illustrating what happened at each step of the factorization.
8. “basis”: The ILR balancing elements constructed from phylofactorization.
9. “ExplainedVar”: The percent of total variance explained by regression on phylofactors (only output for the default `choice="var"`)
10. “nfactors”: the number of factors.
11. “bins”: the resultant bins at the end of the phylofactorization.
12. “bin.sizes”: breakdown of bin sizes by the number of bins with that size.
13. “Monophyletic.clades”: indexes of which bins are monophyletic.

# Summarizing phylofactorization

The “factors” feature summarizes the clades split and how significant these splits were:

```
PF$factors
```

```
##          Group1                         Group2                         
## Factor 1 "40 member Monophyletic clade" "250 member Monophyletic clade"
## Factor 2 "16 member Monophyletic clade" "234 member Paraphyletic clade"
## Factor 3 "15 member Monophyletic clade" "219 member Paraphyletic clade"
##          pvalues
## Factor 1 "0"    
## Factor 2 "0"    
## Factor 3 "0"
```

The first factor splits a 40 member monophyletic clade form a 250 member “monophyletic” clade. Both clades will be monophyletic initially by default as both are monophyletic in an unrooted tree. Researchers can use their own assumptions to re-classify the clades and focus their efforts on monophyletic clades in the rooted tree.

We can look at the regression from each factor through the output glms:

```
PF$glms[[1]]
```

```
## 
## Call:  glm(formula = frmla, data = dataset)
## 
## Coefficients:
## (Intercept)      Xtongue  
##      -5.388       12.872  
## 
## Degrees of Freedom: 39 Total (i.e. Null);  38 Residual
## Null Deviance:       1712 
## Residual Deviance: 54.62     AIC: 132
```

```
summary(aov(PF$glms[[1]]))
```

```
##             Df Sum Sq Mean Sq F value Pr(>F)    
## X            1 1657.0  1657.0    1153 <2e-16 ***
## Residuals   38   54.6     1.4                   
## ---
## Signif. codes:  0 '***' 0.001 '**' 0.01 '*' 0.05 '.' 0.1 ' ' 1
```

Once we have a “phylofactor” object, there are a bunch of functions that start with “pf.” These functions produce some convenient output once you have a phylofactor object.

For instance, we can summarize factor 1 using `pf.summary`:

```
smry <- pf.summary(PF,taxonomy,factor=1)
names(smry)
```

```
## [1] "group1"          "group2"          "TaxaSplit"       "glm"            
## [5] "ilr"             "fitted.values"   "MeanRatio"       "fittedMeanRatio"
```

The summary grabs features about factor 1 that may be of use for downstream analysis and plotting. This includes:

1. “ilr” - the ILR balance for that factor (the dependent variable in the regression)
2. “fitted.values” - the fitted ILR balances from regression
3. “MeanRatio” - the ratio of geometric means of taxa in each group (Group1/Group2)
4. “fittedMeanRatio” - the fitted ratio of geometric means of taxa in each group
5. “TaxaSplit” - a two-member list of the taxonomy of the two groups. The taxonomies are trimmed to the *shortest unique prefix* across groups (that is, the shortest prefix not found in the other group).

To be more succinct, we can input `smry` into another function: `pf.tidy`.

```
pf.tidy(smry)
```

```
## $`group1, Monophyletic`
## [1] k__Bacteria; p__Actinobacteria;                        
## [2] k__Bacteria; p__Proteobacteria; c__Alphaproteobacteria;
## [3] k__Bacteria; p__Proteobacteria; c__Betaproteobacteria; 
## [4] k__Bacteria; p__Proteobacteria; c__Deltaproteobacteria;
## [5] k__Bacteria; p__Proteobacteria; c__Gammaproteobacteria;
## 5 Levels: k__Bacteria; p__Actinobacteria; ...
## 
## $`group2, Monophyletic`
## [1] k__Bacteria; p__Bacteroidetes;                           
## [2] k__Bacteria; p__Cyanobacteria;                           
## [3] k__Bacteria; p__Firmicutes;                              
## [4] k__Bacteria; p__Fusobacteria;                            
## [5] k__Bacteria; p__Proteobacteria; c__Epsilonproteobacteria;
## [6] k__Bacteria; p__Verrucomicrobia;                         
## 6 Levels: k__Bacteria; p__Bacteroidetes; ...
## 
## $`Predicted ratio of group1/group2`
##     feces    tongue 
## 0.3995171 3.5772570 
## 
## $`Observed Ratio of group1/group2 geometric means`
##     feces   feces.1   feces.2   feces.3   feces.4   feces.5   feces.6 
## 0.3932904 0.3691791 0.4240780 0.6664638 0.4614384 0.3368341 0.3867820 
##   feces.7   feces.8   feces.9  feces.10  feces.11  feces.12  feces.13 
## 0.3736680 0.3115788 0.4286058 0.5774729 0.3913563 0.3565216 0.3678096 
##  feces.14  feces.15  feces.16  feces.17  feces.18  feces.19    tongue 
## 0.3923586 0.3938998 0.3749477 0.3672121 0.3785641 0.3651888 4.0964156 
##  tongue.1  tongue.2  tongue.3  tongue.4  tongue.5  tongue.6  tongue.7 
## 4.5410468 3.8226428 4.8166589 3.7904626 4.3919111 4.9287731 4.0662406 
##  tongue.8  tongue.9 tongue.10 tongue.11 tongue.12 tongue.13 tongue.14 
## 3.9905971 4.5949302 3.2815087 3.6910945 3.0107416 2.7560454 2.8664792 
## tongue.15 tongue.16 tongue.17 tongue.18 tongue.19 
## 2.5432438 2.1839945 2.7764474 3.1840250 3.9727941
```

# Visualizing

### phylo.heatmap

Phylogenetic structure in a dataset can often be visualized with phylogenetic heatmaps. I use Liam Revell’s “phytools” package for that. When imaging OTU tables, I find that the centered log-ratio transform often produces better images:

```
library(phytools)
clr <- function(Matrix) apply(Matrix,MARGIN=2,FUN=function(x) log(x)-mean(log(x)))

par(mfrow=c(1,1))
phylo.heatmap(tree,clr(PF$Data))
```

The tip labels and column labels are a bit ugly, but the colormap and alignment of the data with the phylogeny reveal phylogenetic structure in our data as big blocks of color across body sites.

### Abundance differences

What were the differences between Group1 and Group2 in factor 1 above?

```
par(mfrow=c(1,2))
plot(body.site,smry$ilr,main='Isometric log-ratio',ylab='ILR balance')
plot(body.site,smry$MeanRatio,main='Ratio of Geometric Means',ylab='Group1/Group2')
```

```
td <- pf.tidy(smry)
predicted.ratio <- td$`Predicted ratio of group1/group2`
predicted.ratio
```

```
##     feces    tongue 
## 0.3995171 3.5772570
```

```
predicted.ratio['tongue']/predicted.ratio['feces']
```

```
##   tongue 
## 8.953952
```

The ratio of Group1/Group2 changes by a factor of 9 across body sites.

Who are these OTUs being split by this factor? Taxonomies can be obtained using the “TaxaSplit” feature of `smry`:

```
smry$TaxaSplit %>%
      lapply(.,FUN=function(x) unique(x$TaxaIDs))         #this simplifies our list to the unique elements
```

```
## $`Group 1`
## [1] k__Bacteria; p__Actinobacteria;                        
## [2] k__Bacteria; p__Proteobacteria; c__Alphaproteobacteria;
## [3] k__Bacteria; p__Proteobacteria; c__Betaproteobacteria; 
## [4] k__Bacteria; p__Proteobacteria; c__Deltaproteobacteria;
## [5] k__Bacteria; p__Proteobacteria; c__Gammaproteobacteria;
## 5 Levels: k__Bacteria; p__Actinobacteria; ...
## 
## $`Group 2`
## [1] k__Bacteria; p__Bacteroidetes;                           
## [2] k__Bacteria; p__Cyanobacteria;                           
## [3] k__Bacteria; p__Firmicutes;                              
## [4] k__Bacteria; p__Fusobacteria;                            
## [5] k__Bacteria; p__Proteobacteria; c__Epsilonproteobacteria;
## [6] k__Bacteria; p__Verrucomicrobia;                         
## 6 Levels: k__Bacteria; p__Bacteroidetes; ...
```

Group 1 contains Actinobacteria and all classes of Proteobacteria except Epsilonproteobacteria.

We can summarize our first factor as follows: the Actinobacteria & all non-Epsilon-Proteobacteria appear to be tongue specialists. On average, they are 0.4x as abundant as other taxa in the gut, whereas in the tongue they are on average 3.6x as abundant as other taxa.

### Mapping phylofactors to phylogeny

Each factor corresponds to a chain of one or more edges in the phylogeny. The function `getFactoredEdges` helps us find out which edges those are:

```
factored.edge <- getFactoredEdges(PF$basis[,1],tree) 
factored.edges <- getFactoredEdgesPAR(ncores=3,PF=PF) #Parallel; gets all factored edges
```

With the factored edges, we can map our phylofactors directly to the phylogeny. In the future, this may help with annotation; for now, it helps with visualization. Let’s color the edges for each group, and exaggerate the length of the factored edge:

```
Group1.otus <- smry$group1$IDs$otuIDs %>% as.list %>% sapply(.,toString)
Group2.otus <- smry$group2$IDs$otuIDs %>% as.list %>% sapply(.,toString)
Group1.edges <- extractEdges(tree,taxa=Group1.otus,type=3)
Group2.edges <- extractEdges(tree,taxa=Group2.otus,type=3)

edge.colors <- rep('black',Nedge(tree))
edge.colors[Group1.edges] <- 'green'
edge.colors[Group2.edges] <- 'blue'
edge.colors[factored.edge] <- 'red'

### Let's also exaggerate the length of our factored edge:
edge.lengths <- tree$edge.length
edge.lengths[factored.edge] <- 0.6

tr <- tree
tr$edge.length <- edge.lengths
par(mfrow=c(1,1))
plot.phylo(tr,type='unrooted',edge.color = edge.colors,show.tip.label = F,edge.width = 2,main='Factor 1',rotate.tree = 20)

legend(-0.16,0.3,legend=c('Group 1','factored edge','Group 2'),fill=c('blue','red','green'))
```

### ILR Ordination

Phylofactorization is a change of variables that allows for dimensionality reduction and ordination visualization.

```
pf.ordination(PF)
```

This ordination-visualization is very easy to do yourself, and doing it yourself can provide more flexibility. To do it yourself, just project the data onto ILR coordinates and choose which coordinates to plot.

```
ilr.projection <- pf.ILRprojection(PF,nfactors=2)
plot(ilr.projection[1,],ilr.projection[2,],xlab='PF1',ylab='PF2',main='Ordination by ILR Coordinates')
points(ilr.projection[1,body.site=='feces'],ilr.projection[2,body.site=='feces'],pch=16,cex=2,col='brown')
points(ilr.projection[1,body.site=='tongue'],ilr.projection[2,body.site=='tongue'],pch=16,cex=2,col='pink')
legend(-1,2.5,legend=c('Tongue','Poo'),fill=c('pink','brown'),cex=2)
```

### Bin Projection

We can bin taxa based on their common factors at a given level of factorization, forming binned phylogenetic units or BPUs.

```
bin.projection <- pf.BINprojection(PF,factor=3)
```

The default `pf.BINprojection` returns the observed geometric means of the relative abundances of taxa in each bin.

We can also obtain the predicted geometric means of taxa in each bin using the input `prediction`

```
bin.projection.pred <- pf.BINprojection(PF,prediction=T)
names(bin.projection.pred)
```

```
## [1] "Data" "otus"
```

The output from `pf.Binprojection` contains the “Data” matrix and the “otus” in each bin.

The taxonomic composition of bins can be summarized with help from the function `OTUtoTaxa`:

```
bin.taxa <- bin.projection$otus %>% 
              lapply(.,FUN=function(otus,tax) OTUtoTaxa(otus,tax,common.name = F),tax=taxonomy)
bin.taxa[2:4]
```

```
## $`Bin 2`
##  [1] "k__Bacteria; p__Actinobacteria; c__Actinobacteria; o__Actinomycetales; f__Actinomycetaceae; g__; s__"                            
##  [2] "k__Bacteria; p__Actinobacteria; c__Actinobacteria; o__Actinomycetales; f__Actinomycetaceae; g__Actinomyces; s__"                 
##  [3] "k__Bacteria; p__Actinobacteria; c__Actinobacteria; o__Actinomycetales; f__Actinomycetaceae; g__Actinomyces; s__"                 
##  [4] "k__Bacteria; p__Actinobacteria; c__Actinobacteria; o__Actinomycetales; f__Actinomycetaceae; g__Arcanobacterium; s__"             
##  [5] "k__Bacteria; p__Actinobacteria; c__Actinobacteria; o__Actinomycetales; f__Corynebacteriaceae; g__Corynebacterium; s__"           
##  [6] "k__Bacteria; p__Actinobacteria; c__Actinobacteria; o__Actinomycetales; f__Micrococcaceae; g__Rothia; s__aeria"                   
##  [7] "k__Bacteria; p__Actinobacteria; c__Actinobacteria; o__Actinomycetales; f__Micrococcaceae; g__Rothia; s__mucilaginosa"            
##  [8] "k__Bacteria; p__Actinobacteria; c__Actinobacteria; o__Actinomycetales; f__Micrococcaceae; g__Rothia; s__mucilaginosa"            
##  [9] "k__Bacteria; p__Actinobacteria; c__Coriobacteriia; o__Coriobacteriales; f__Coriobacteriaceae; g__Atopobium; s__"                 
## [10] "k__Bacteria; p__Proteobacteria; c__Alphaproteobacteria; o__Rickettsiales; f__mitochondria; g__; s__"                             
## [11] "k__Bacteria; p__Proteobacteria; c__Betaproteobacteria; o__Burkholderiales; f__Alcaligenaceae; g__Sutterella; s__"                
## [12] "k__Bacteria; p__Proteobacteria; c__Betaproteobacteria; o__Burkholderiales; f__Burkholderiaceae; g__Lautropia; s__"               
## [13] "k__Bacteria; p__Proteobacteria; c__Betaproteobacteria; o__Burkholderiales; f__Burkholderiaceae; g__Lautropia; s__"               
## [14] "k__Bacteria; p__Proteobacteria; c__Betaproteobacteria; o__Burkholderiales; f__Comamonadaceae; g__; s__"                          
## [15] "k__Bacteria; p__Proteobacteria; c__Betaproteobacteria; o__Neisseriales; f__Neisseriaceae; g__; s__"                              
## [16] "k__Bacteria; p__Proteobacteria; c__Betaproteobacteria; o__Neisseriales; f__Neisseriaceae; g__Eikenella; s__"                     
## [17] "k__Bacteria; p__Proteobacteria; c__Betaproteobacteria; o__Neisseriales; f__Neisseriaceae; g__Neisseria; s__"                     
## [18] "k__Bacteria; p__Proteobacteria; c__Betaproteobacteria; o__Neisseriales; f__Neisseriaceae; g__Neisseria; s__"                     
## [19] "k__Bacteria; p__Proteobacteria; c__Betaproteobacteria; o__Neisseriales; f__Neisseriaceae; g__Neisseria; s__cinerea"              
## [20] "k__Bacteria; p__Proteobacteria; c__Betaproteobacteria; o__Neisseriales; f__Neisseriaceae; g__Neisseria; s__subflava"             
## [21] "k__Bacteria; p__Proteobacteria; c__Betaproteobacteria; o__Neisseriales; f__Neisseriaceae; g__Neisseria; s__subflava"             
## [22] "k__Bacteria; p__Proteobacteria; c__Deltaproteobacteria; o__Desulfovibrionales; f__Desulfovibrionaceae; g__Bilophila; s__"        
## [23] "k__Bacteria; p__Proteobacteria; c__Deltaproteobacteria; o__Desulfovibrionales; f__Desulfovibrionaceae; g__Desulfovibrio; s__"    
## [24] "k__Bacteria; p__Proteobacteria; c__Gammaproteobacteria; o__Cardiobacteriales; f__Cardiobacteriaceae; g__Cardiobacterium; s__"    
## [25] "k__Bacteria; p__Proteobacteria; c__Gammaproteobacteria; o__Cardiobacteriales; f__Cardiobacteriaceae; g__Cardiobacterium; s__"    
## [26] "k__Bacteria; p__Proteobacteria; c__Gammaproteobacteria; o__Enterobacteriales; f__Enterobacteriaceae; g__; s__"                   
## [27] "k__Bacteria; p__Proteobacteria; c__Gammaproteobacteria; o__Enterobacteriales; f__Enterobacteriaceae; g__Serratia; s__"           
## [28] "k__Bacteria; p__Proteobacteria; c__Gammaproteobacteria; o__Pasteurellales; f__Pasteurellaceae; g__Actinobacillus; s__porcinus"   
## [29] "k__Bacteria; p__Proteobacteria; c__Gammaproteobacteria; o__Pasteurellales; f__Pasteurellaceae; g__Aggregatibacter; s__"          
## [30] "k__Bacteria; p__Proteobacteria; c__Gammaproteobacteria; o__Pasteurellales; f__Pasteurellaceae; g__Haemophilus; s__parainfluenzae"
## [31] "k__Bacteria; p__Proteobacteria; c__Gammaproteobacteria; o__Pasteurellales; f__Pasteurellaceae; g__Haemophilus; s__parainfluenzae"
## [32] "k__Bacteria; p__Proteobacteria; c__Gammaproteobacteria; o__Pasteurellales; f__Pasteurellaceae; g__Haemophilus; s__parainfluenzae"
## [33] "k__Bacteria; p__Proteobacteria; c__Gammaproteobacteria; o__Pasteurellales; f__Pasteurellaceae; g__Haemophilus; s__parainfluenzae"
## [34] "k__Bacteria; p__Proteobacteria; c__Gammaproteobacteria; o__Pasteurellales; f__Pasteurellaceae; g__Haemophilus; s__parainfluenzae"
## [35] "k__Bacteria; p__Proteobacteria; c__Gammaproteobacteria; o__Pasteurellales; f__Pasteurellaceae; g__Haemophilus; s__parainfluenzae"
## [36] "k__Bacteria; p__Proteobacteria; c__Gammaproteobacteria; o__Pasteurellales; f__Pasteurellaceae; g__Haemophilus; s__parainfluenzae"
## [37] "k__Bacteria; p__Proteobacteria; c__Gammaproteobacteria; o__Pasteurellales; f__Pasteurellaceae; g__Haemophilus; s__parainfluenzae"
## [38] "k__Bacteria; p__Proteobacteria; c__Gammaproteobacteria; o__Pasteurellales; f__Pasteurellaceae; g__Haemophilus; s__parainfluenzae"
## [39] "k__Bacteria; p__Proteobacteria; c__Gammaproteobacteria; o__Pseudomonadales; f__Pseudomonadaceae; g__Pseudomonas; s__"            
## [40] "k__Bacteria; p__Proteobacteria; c__Gammaproteobacteria; o__Pseudomonadales; f__Pseudomonadaceae; g__Pseudomonas; s__"            
## 
## $`Bin 3`
##  [1] "k__Bacteria; p__Firmicutes; c__Bacilli; o__Gemellales; f__Gemellaceae; g__Gemella; s__"                  
##  [2] "k__Bacteria; p__Firmicutes; c__Bacilli; o__Lactobacillales; f__Carnobacteriaceae; g__; s__"              
##  [3] "k__Bacteria; p__Firmicutes; c__Bacilli; o__Lactobacillales; f__Carnobacteriaceae; g__Carnobacterium; s__"
##  [4] "k__Bacteria; p__Firmicutes; c__Bacilli; o__Lactobacillales; f__Streptococcaceae; g__Lactococcus; s__"    
##  [5] "k__Bacteria; p__Firmicutes; c__Bacilli; o__Lactobacillales; f__Streptococcaceae; g__Streptococcus; s__"  
##  [6] "k__Bacteria; p__Firmicutes; c__Bacilli; o__Lactobacillales; f__Streptococcaceae; g__Streptococcus; s__"  
##  [7] "k__Bacteria; p__Firmicutes; c__Bacilli; o__Lactobacillales; f__Streptococcaceae; g__Streptococcus; s__"  
##  [8] "k__Bacteria; p__Firmicutes; c__Bacilli; o__Lactobacillales; f__Streptococcaceae; g__Streptococcus; s__"  
##  [9] "k__Bacteria; p__Firmicutes; c__Bacilli; o__Lactobacillales; f__Streptococcaceae; g__Streptococcus; s__"  
## [10] "k__Bacteria; p__Firmicutes; c__Bacilli; o__Lactobacillales; f__Streptococcaceae; g__Streptococcus; s__"  
## [11] "k__Bacteria; p__Firmicutes; c__Bacilli; o__Lactobacillales; f__Streptococcaceae; g__Streptococcus; s__"  
## [12] "k__Bacteria; p__Firmicutes; c__Bacilli; o__Lactobacillales; f__Streptococcaceae; g__Streptococcus; s__"  
## [13] "k__Bacteria; p__Firmicutes; c__Bacilli; o__Lactobacillales; f__Streptococcaceae; g__Streptococcus; s__"  
## [14] "k__Bacteria; p__Firmicutes; c__Bacilli; o__Lactobacillales; f__Streptococcaceae; g__Streptococcus; s__"  
## [15] "k__Bacteria; p__Firmicutes; c__Bacilli; o__Lactobacillales; f__Streptococcaceae; g__Streptococcus; s__"  
## [16] "k__Bacteria; p__Firmicutes; c__Bacilli; o__Lactobacillales; f__Streptococcaceae; g__Streptococcus; s__"  
## 
## $`Bin 4`
##  [1] "k__Bacteria; p__Bacteroidetes; c__Bacteroidia; o__Bacteroidales; f__Prevotellaceae; g__Prevotella; s__"              
##  [2] "k__Bacteria; p__Bacteroidetes; c__Bacteroidia; o__Bacteroidales; f__Prevotellaceae; g__Prevotella; s__"              
##  [3] "k__Bacteria; p__Bacteroidetes; c__Bacteroidia; o__Bacteroidales; f__Prevotellaceae; g__Prevotella; s__"              
##  [4] "k__Bacteria; p__Bacteroidetes; c__Bacteroidia; o__Bacteroidales; f__Prevotellaceae; g__Prevotella; s__"              
##  [5] "k__Bacteria; p__Bacteroidetes; c__Bacteroidia; o__Bacteroidales; f__Prevotellaceae; g__Prevotella; s__"              
##  [6] "k__Bacteria; p__Bacteroidetes; c__Bacteroidia; o__Bacteroidales; f__Prevotellaceae; g__Prevotella; s__"              
##  [7] "k__Bacteria; p__Bacteroidetes; c__Bacteroidia; o__Bacteroidales; f__Prevotellaceae; g__Prevotella; s__"              
##  [8] "k__Bacteria; p__Bacteroidetes; c__Bacteroidia; o__Bacteroidales; f__Prevotellaceae; g__Prevotella; s__"              
##  [9] "k__Bacteria; p__Bacteroidetes; c__Bacteroidia; o__Bacteroidales; f__Prevotellaceae; g__Prevotella; s__"              
## [10] "k__Bacteria; p__Bacteroidetes; c__Bacteroidia; o__Bacteroidales; f__Prevotellaceae; g__Prevotella; s__copri"         
## [11] "k__Bacteria; p__Bacteroidetes; c__Bacteroidia; o__Bacteroidales; f__Prevotellaceae; g__Prevotella; s__melaninogenica"
## [12] "k__Bacteria; p__Bacteroidetes; c__Bacteroidia; o__Bacteroidales; f__Prevotellaceae; g__Prevotella; s__melaninogenica"
## [13] "k__Bacteria; p__Bacteroidetes; c__Bacteroidia; o__Bacteroidales; f__Prevotellaceae; g__Prevotella; s__melaninogenica"
## [14] "k__Bacteria; p__Bacteroidetes; c__Bacteroidia; o__Bacteroidales; f__Prevotellaceae; g__Prevotella; s__nanceiensis"   
## [15] "k__Bacteria; p__Bacteroidetes; c__Bacteroidia; o__Bacteroidales; f__Prevotellaceae; g__Prevotella; s__nanceiensis"
```

The bins can be stacked to visualize their relative weight or abundances. The default output for `pf.BINprojection` will be a data matrix whose rows are the *geometric means* of the bins. The input `rel.abund` allows us to grab the observed and predicted *relative abundances* as well:

```
bin.observed.geometricMeans <- pf.BINprojection(PF)$Data
bin.observed.relativeAbundances <- pf.BINprojection(PF,rel.abund=T)$Data
bin.predicted.geometricMeans <- pf.BINprojection(PF,prediction=T)$Data
bin.predicted.relativeAbundances <- pf.BINprojection(PF,prediction=T,rel.abund=T)$Data

library(plotrix)
par(mfrow=c(2,2))
stackpoly(t(bin.observed.geometricMeans),stack=T,main='Observed gMean',xat = c(10,30),xaxlab = c('feces','tongue'))
stackpoly(t(bin.predicted.geometricMeans),stack=T,main='Predicted gMean',xat = c(10,30),xaxlab = c('feces','tongue'))

stackpoly(t(bin.observed.relativeAbundances),stack=T,main='Observed Rel-Abund.',xat = c(10,30),xaxlab = c('feces','tongue'))
stackpoly(t(bin.predicted.relativeAbundances),stack=T,main='Predicted Rel-Abund.',xat = c(10,30),xaxlab = c('feces','tongue'))
```

# Make predictions with phylofactor

The generalized linear models in the default phylofactorization can be used to make predictions. Like the `predict` function for regular “glm” objects, `pf.predict` combines the glms to predict the relative abundances of OTUs.

```
prediction <- pf.predict(PF)
dim(prediction)
```

```
## [1] 290  40
```

For user flexibility, `pf.predict` returns an entire data matrix.

We can visualize the Data and predictions side-by-side:

```
prediction <- pf.predict(PF,factors=3)
par(mfrow=c(2,1))
phylo.heatmap(tree,clr(PF$Data))
phylo.heatmap(tree,clr(prediction))
```

```
## Warning: closing unused connection 7 (<-LAPTOP-T9KHGO63:11489)
```

```
## Warning: closing unused connection 6 (<-LAPTOP-T9KHGO63:11489)
```

```
## Warning: closing unused connection 5 (<-LAPTOP-T9KHGO63:11489)
```

# Alternative objective function

Which edge is “best”? Previously, it was the edge that “explained the most variance”, but `PhyloFactor` has an additional objective function for regression-phylofactorization.

The default objective function picks out the ILR basis element whose balance has the largest difference between null and residual deviance. The default objective function captures and explains the most variance, but what if our objective is different?

For instance, what if, instead of “capturing total variance”, we want to know which clades are most predictable i.e. have the largest ratio of explained to unexplained variance? The former objective function explains variation in communities, whereas the latter may be better at finding bio-indicators and clades most likely to have functional ecological relationships with the independent variables, even if their responses don’t capture much of the variance in the community.

To ensure everyone is on the same page, let’s illustrate the distinction between these two objective functions by simulation. We’ll simulate two random variables, one which will have more explained variance and one which is ‘more predictable’:

```
set.seed(1)
x <- rnorm(100)
y1 <- 10*x+10*rnorm(100)   #This dependent variable has more variance that we can capture
y2 <- 5*x +2*rnorm(100) #This dependent variable has less variance to capture but a more predictable signal:

glm.1 <- glm(y1~x)
glm.2 <- glm(y2~x)

par(mfrow=c(1,2))
plot(x,y1,ylim=c(-40,40),main='More Var Explained')
lines(x,glm.1$fitted.values)
plot(x,y2,ylim=c(-40,40),main='More Predictable')
lines(x,glm.2$fitted.values)
```

The regression in the first plot explains more variance, since `y1` is more variable. The second regression on `y2`, however, is more predictable - the ratio of explained to unexplained variance is much higher.

In `PhyloFactor`, these statistics are computed using the `getStats` function:

```
getStats(glm.1)
```

```
##         Pval            F ExplainedVar 
## 4.662937e-15 8.598627e+01 7.970015e+01
```

```
getStats(glm.2)
```

```
##         Pval            F ExplainedVar 
##      0.00000    469.95626     20.30691
```

The P-values are computed from an F test. Notice that the first regression has a lower F-statistic but a higher Explained Variance. Conversely, the second regression has a higher F-statistic but a lower Explained Variance.

To phylofactor based on F-statistics, input to PhyloFactor `choice='F'`.

```
PF.F <- PhyloFactor(OTUTable,tree,body.site,choice='F',nfactors=3)
```

# Multiple Regression

With the `glm` function at the heart of `PhyloFactor`, we can also use more flexible regression models to phylofactor our data. Suppose we also measure age and want to know which edges capture most of the variation in OTU abundances due to both `body.site` and `age`. Let’s simulate some ages:

```
age <- rpois(40,lambda=50)
```

To phylofactor the data based on multiple regression of body site and age, we need input much like that of `glm`:

1. a formula for our regression and
2. a data frame containing all the independent variables.

Phylofactor inputs the formula straight into `glm()`, so we can look at the logarithm of the age or other standard formulas that `glm()` accepts. One catch: when making your formula, *you must have the ILR balances, in this case the dependent variable, labelled “Data”*.

```
frmla <- Data ~ body.site+log(age) #Must put ILR coordinates as "Data"
my.data.frame <- data.frame('body.site'=body.site,'age'=age)
```

Phylofactorization from multiple regression is as easy as:

```
PF.mult <- PhyloFactor(OTUTable,tree,X=my.data.frame,frmla=frmla,nfactors=3)
```

As before, we have glms in our phylofactor object, but now the glms capture our multiple regression and can be used for downstream analysis (e.g. dominance analysis, as we did in our paper).

```
PF.mult$glms[[1]]
```

```
## 
## Call:  glm(formula = frmla, data = dataset)
## 
## Coefficients:
##     (Intercept)  body.sitetongue         log(age)  
##         -8.4795          12.8369           0.7992  
## 
## Degrees of Freedom: 39 Total (i.e. Null);  37 Residual
## Null Deviance:       1712 
## Residual Deviance: 54.19     AIC: 133.7
```

```
summary(aov(PF.mult$glms[[1]]))
```

```
##             Df Sum Sq Mean Sq  F value Pr(>F)    
## body.site    1 1657.0  1657.0 1131.327 <2e-16 ***
## log(age)     1    0.4     0.4    0.291  0.593    
## Residuals   37   54.2     1.5                    
## ---
## Signif. codes:  0 '***' 0.001 '**' 0.01 '*' 0.05 '.' 0.1 ' ' 1
```

# Parallelization

Phylofactorization of big OTU tables is computationally intensive, but can be parallelized through the input `ncores`. It’s as easy as that:

```
PF.par <- PhyloFactor(OTUTable,tree,body.site,nfactors=3,ncores=7)
all.equal(PF,PF.par)
```

```
## [1] TRUE
```

Parallelization is done with a PSOCK cluster to which we export the needed functions and libraries (this is done with the function `phyloFcluster` if you want to rig up your own). The percent of the code that is parellelizable increases with the size of the dataset. When parellelizing phylofactor, watch your RAM use - all workers will need to work with at least one copy of the dataset, plus some extra memory for temporary variables. This can lead to high memory requirements for big datasets sent to many workers.

# Guts of PhyloFactor and Advanced Ninja S\*\*\*

PhyloFactor is a complex algorithm with more generality and flexibility than the default settings. In this section, we’ll talk about the guts of phylofactor so that a well-informed user/ninja can better manipulate PhyloFactor to fit their questions and model assumptions.

### Phylofactorization for Geeks/Ninjas

Phylofactorization is a matrix factorization, a change of basis from the elementary basis to one with a useful, phylogenetic interpretation of our data.

Instead of using basis elements like (1,0,0,0) - the abundances of species 1 - (0,1,0,0) - the abundance of species 2 - and so on, we can use basis elements such as (1,1,-1,-1) - the difference in abundance of species 1 and 2 from species 3 and 4 - and (1,-1,0,0) - the difference in abundance of species 1 and 2. Since any edge in a phylogeny (in fact any edge in any graph without cycles) splits the species in the community in two, each edge has its own natural “basis element” corresponding to the difference between taxa on each side of the edge.

Phylofactorization, in its current form, is built for compositional data. In compositional data, the vector (1,1,1,1) already has a fixed coordinate: 1. Since species relative abundances sum to 1, this vector, (1,1,1,1) is uninteresting. To construct more interesting vectors, they should be orthogonal to (1,1,1,1) and correspond to egdes in our tree. Let’s make a demo tree to illustrate this:

```
library(ape)
set.seed(4)
demo.tree <- rtree(4)
par(mfrow=c(1,1))
plot.phylo(demo.tree,use.edge.length = F,show.tip.label = F,main='Demo Phylogeny')
tiplabels(cex=2)
edgelabels(cex=2)
```

To construct basis elements orthogonal to (1,1,1,1), we can do the following.

For edge 1 in the figure above, we can try the basis element (1,1,-1,-1). The coordinate from projecting the data onto this basis element carries information about the difference between the group {1,2} and the group {3,4}: if this coordinate increases, then we know {1,2} go up relative to {3,4}, and vice versa if this coordinate decreases. Notice: edge 4 carries the same information, so we can neglect it. This is not problematic, because if we infer that {1,2} is different from {3,4}, we don’t know if a putative trait undelrying this difference arose along edge 1 or edge 4.

For edge 2, the basis element (3,-1,-1,-1) can be considered.

Suppose our first iteration identifies edge 1 as the ‘most interesting’ edge. Now, we want to consider basis elements that are orthogonal to (1,1,1,1) AND (1,1,-1,-1). For edge 2, that would be (1,-1,0,0) (and now edge 3 is equivalent to edge 2).

Now, a few details arise. First, and easiest to understand, is that we need to normalize those vectors above so that they have unit norm. Thus, the vector (1,1,-1,-1) should really be 0.5\*(1,1,-1,-1), etc. This puts all the edges on a level playing field. It also makes the edge more easily interpretable as the difference between means of taxa in each group.

Second, for compositional data addition and subtraction are not the best operations for finding centers (e.g. the mean of a set of abundances) and distances (e.g. the difference between means); instead, we take products and ratios (for a contemporary textbook on compositional data analysis, see “Modelling and Analysis of Compositional Data” by Vera Pawlowsky-Glahn et al.). Thankfully, if we take logarithms of our data, then we can add and subtract the logarithms and use all the intuition we generated above. If we take the logarithm of a compositional vector then mean-center it, we’ve invented the centered-log ratio (CLR). If we project that CLR vector onto, say, (0.5,0.5,-0.5,-0.5), then we get an “isometric log-ratio” (ILR) “balance”, where (0.5,0.5,-0.5,-0.5) is the “balancing element”. The terms are confusing but the concepts aren’t: we’re looking at a measure of the difference between the means of two disjoint groups of taxa on each side of an edge.

That’s phylofactorization: we log-transform our data, project it onto a whole bunch of basis elements that correspond to edges in the tree, and then choose the “best” edge based on some feature of the edge’s observed balances.

The default ways for choosing the “best” edge are centered on generalized linear modelling of the balances, what I like to call ‘regression-phylofactorization’, which we talk about next. However, if you’re looking beyond regression, `PhyloFactor` permits users to interact with the balances to choose your own “best” edges, thereby permitting more general criteria for this phylogenetically-informed matrix factorization.

### Generalized Linear Modelling in PhyloFactor

At every iteration of `PhyloFactor`, we construct ILR balances, one for each edge under consideration, and have to choose which one is “the best”. Due to the explosion of sequence-count data trying to look at correlations between microbial abundances and various meta-data (disease state, diet, ecosystem function, etc.), the default way of choosing “the best” is based on regression, and that regression is done with the `glm` function.

The default formula for `PhyloFactor` is `frmla=Data~X` where “Data” will end up being each one of our ILR balances and “X” is our independent variable. We’ve already shown how this can be modified to perform multiple regression, but it’s worth pointing out that the optional arguments of PhyloFactor, `...`, are input into `glm`. Thus, deep down in the guts of PhyloFactor is:

`glm(frmla,dataset,...)`

The ILR balances are assumed to be Gaussian and `PhyloFactor` aims to predict these assumed-Gaussian balances using the independent variables, `X`.

For big data with many samples, one can also use the `bigglm` function by passing the input `smallglm=FALSE` to `PhyloFactor`. This reduces the memory costs of the big data, but, if concerned about memory, the user can use methods to trim the output from `glm` with optional arguments such as `y=FALSE`, `model=FALSE`, and other ninja tricks.

Because PhyloFactor works with `glm` and the optional arguments for PhyloFactor go to `glm`, you have some flexibility in how you do regression. If we’re interested in age classification, for instance, we don’t want to know which edge is *best predicted by* the age, but which edge *best predicts* the age. If our age data are Poisson, we can input the optional argument `family='poisson'` to `glm` and phylofactor accordingly.

```
age <- data.frame('age'=rpois(ncol(OTUTable),lambda=50))
PF.age <- PhyloFactor(OTUTable,tree,X=age,frmla='age~Data',family='poisson',nfactors=2)
PF.age$glms[[1]]
```

```
## 
## Call:  glm(formula = frmla, family = "poisson", data = dataset)
## 
## Coefficients:
## (Intercept)         Data  
##     3.91722      0.03782  
## 
## Degrees of Freedom: 39 Total (i.e. Null);  38 Residual
## Null Deviance:       28.65 
## Residual Deviance: 24.92     AIC: 259.6
```

### Objective Functions

The objective functions used are defined in the input `choice` in `PhyloFactor`. They can either be `choice='var'` or `choice='F'`. These statistics are calculated from either `glm` or `bigglm` objects in the function `getStats`.

The `choice='var'` is the default and is computed as the difference between the null and residual deviance. The `choice='F'` is computed as the F statistic from an analysis of variance of the regression model. See `getStats` for the exact details.

### Stop function

What are the criteria by which we should stop phylofactorization? We expect to see ongoing research in this question, and so we have provided both a default stopping function and a way to input your own customized stopping function.

Our default stopping function relies on a Kolmogorov-Smirnov test of the uniformity of P-values from the regressions (specifically, from the F-tests performed in getStats). If the KS test yields `P>KS.Pthreshold`, the factorization is terminated. There are three inputs to PhyloFactor that allow the user to control the termination of `PhyloFactor`: `stop.fcn`, `stop.early` and `KS.Pthreshold`.

The default settings are `stop.fcn=NULL`, `stop.early=NULL`, and `KS.Pthreshold=0.01`.

Inputs to `stop.fcn` can be either the string, ‘KS’, or an actual function of your choice (described below in “customizing phylofactor”).

The input `stop.early` is a logical; if `TRUE`, PhyloFactor will calculate the `stop.fcn` and, if `stop.fcn` tells the simulation to stop, PhyloFactor will stop before adding the latest factor to our phylofactor object. If `stop.early=FALSE`, PhyloFactor will “stop late” and generously add the latest factor before terminating the factorization.

### Customized PhyloFactorization

Now you know the gist of how to customize phylofactor: there are balances that correspond to edges, and you can input a `choice.fcn` and `stop.fcn` to perform your own, customized phylofactorization.

The `choice.fcn` is a function that must take as input:

1. `y`, the ILR balance
2. `X`, input to PhyloFactor under the same name
3. `PF.output`, logical controlling the output of `choice.fcn`. If `TRUE`, the output will be put directly into our “phylofactor” object under `$custom.output`.
4. `...` optional arguments.

The output of `choice.fcn` for `PF.output=FALSE` must be a named list containing:

1. `"objective"` Numeric. The edge with the maximum “objective” will be chosen as the factor.
2. `"stopStatistics"` Whatever you want. These will be input as a list into the `stop.fcn` to allow customized stopping criteria.

For parallelization of a `choice.fcn` with dependencies, the dependencies need to be specified with the `PhyloFactor` input `choice.fcn.dependencies` (described below).

Let’s see an example of how to customize `PhyloFactor` to perform multivariate generalized additive modelling using a trimmed (but real-ish) tree from FTmicrobiome.

```
library(phangorn)
set.seed(1)
tree <- FTmicrobiome$tree
tree <- drop.tip(tree,setdiff(tree$tip.label,sample(tree$tip.label,20)))
```

First, let’s simulate some data. We’ll pick two edges as factors with a known effect:

```
Factornodes <- c(37,31)
sigClades <- Descendants(tree,Factornodes,type='tips')
Factoredges <- sapply(Factornodes,FUN=function(n,tree) which(tree$edge[,2]==n),tree=tree)
par(mfrow=c(1,1))
plot.phylo(tree,use.edge.length=F,main='Community Phylogeny')
edgelabels(c('PF 1','PF 2'),edge=Factoredges,cex=1.2,bg='red')
```

Second, we create the relative-abundance matrix and two independent variables in our data frame, `X`.

```
set.seed(1.1)
n=100
Data <- matrix(rlnorm(20*n,meanlog = 8,sdlog = .5),nrow=20)
rownames(Data) <- tree$tip.label
a <- rnorm(n)
b <- rnorm(n)
X <- data.frame(a,b)
```

We’re going to give non-linear dependence of species’ abundances on the independent variables, `a` and `b` for species in our two clades. The first clade decreases nonlinearly with `b`. The second clade is abundant only for intermediate values of `a`.

```
Data[sigClades[[1]],] <- t(t(Data[sigClades[[1]],])*(20/(1+exp(5*b)))) 
## This clade has a nonlinear response with b, decreasing for high values of b.

Data[sigClades[[2]],] <- t(t(Data[sigClades[[2]],])*8*exp(-4*(a)^2)) 
## this clade is abundant only for intermediate values of a.

clo <- function(A) apply(A,MARGIN=2,FUN=function(x) x/sum(x)) #convert to relative abundances
geomean <- function(A) apply(A,MARGIN=2,FUN=function(x) exp(mean(log(x))))
Data <- clo(Data)
 
par(mfrow=c(1,2))
plot(b,geomean(Data[sigClades[[1]],]),ylab="First Clade's gMean")
plot(a,geomean(Data[sigClades[[2]],]),ylab="Second Clade's gMean")
```

Next, we have to define a customized `choice.fcn`. Our choice function will perform generalized additive modelling from the package `mgcv`. The `objective` will be the difference between the null and residual deviance and the `stopStatistics` will be the P-value from an F-test in getStats.

```
GAM <- function(y,X,PF.output=F,...){
   dataset <- cbind(y,X)
   gg <- mgcv::gam(y~s(a)+s(b),data=dataset,...)
 
   if (PF.output){ ## this is what we output into our "phylofactor" object
     return(gg) #return our generalized additive model
     break
   } else { ## this is what we output into PhyloFactor to find the best edge
     output <- NULL
     output$objective <- getStats(gg)['ExplainedVar'] #used to find best edge
     output$stopStatistics <- getStats(gg)['Pval']     #used for stop.fcn
     return(output)
   }
}
```

Since our `choice.fcn` depends on the package `mgcv`, we need to define our `choice.fcn.dependencies` function which loads or defines all the stuff needed for our `choice.fcn`. This function will be passed to all of the workers in our PSOCK cluster to ensure they have what they need to use our custom `choice.fcn`.

```
choice.fcn.dependency <- function() library(mgcv)
```

Lastly, we’ll define our `stop.fcn`. The `stop.fcn` must take as input our `stopStatistics` defined above and output a logical indicating whether or not to stop (`TRUE` will stop phylofactorization).

Let’s make a `stop.fcn` based on a minimum P-value and a Bonferroni correction as an attempt to control the family-wide error rate over all the edges (word of caution: this `stop.fcn` is illustrative but not proven to give a well-defined family-wide error rate for phylofactor. Maybe you can make that paper?).

```
Bonferonni.stopper <- function(Pvals){
  Pvals <- unlist(Pvals) ## The stopStatistics will be in a list
  if (min(Pvals) > 0.05/length(Pvals)){
    return(TRUE)
  } else {
    return(FALSE)
  }
}
```

We’re all set. We can now phylofactor the data based on our custom objective function whose dependencies will be exported to the workers, stop the factorization with our custom stopping function, and do it all with internal parallelization:

```
PF.gam <- PhyloFactor(Data,tree,X,choice.fcn = GAM, choice.fcn.dependencies = choice.fcn.dependency, ncores=3,stop.fcn=Bonferonni.stopper,stop.early=T)

sigClades
```

```
## [[1]]
## [1] 15 16
## 
## [[2]]
## [1] 6 7 8
```

```
PF.gam$bins[2:3] #pulled out from phylofactorization
```

```
## $Monophyletic
## [1] 6 7 8
## 
## $Monophyletic
## [1] 15 16
```

```
all(sigClades %in% PF.gam$bins)
```

```
## [1] TRUE
```

This phylofactor object contains our `$custom.output` from our `choice.fcn` when `PF.output=TRUE`:

```
PF.gam$custom.output[[1]]
```

```
## 
## Family: gaussian 
## Link function: identity 
## 
## Formula:
## y ~ s(a) + s(b)
## 
## Estimated degrees of freedom:
## 8.00 2.92  total = 11.92 
## 
## GCV score: 0.2959702
```

And now we can do whatever we want with it!

```
par(mfrow=c(1,2))
 for (ff in 1:2){
   gm <- PF.gam$custom.output[[ff]]
   grp <- PF.gam$groups[[ff]]
   if (ff==2){
     x=b
     nd <- X
     nd$a <- rep(mean(X$a),length(X$a))
   } else {
     x=X$a
     nd <- X
     nd$b <- rep(mean(X$b),length(X$b))
   }
   
   y <- amalg.ILR(grp,log(Data))    #observed ILR coordinates
   pred <- predict(gm,newdata = nd) #predicted ILR coordinates
   plot(sort(x),y[order(x)],ylab='ILR Coefficient',xlab='dominant Independent    Variable',main=paste('Factor',toString(ff),sep=' '))
   lines(sort(x),pred[order(x)])
 }
```

# The end!

That’s all for now, folks! I need a beer.

For more functionality of the phylofactor package, try the `?` command - each function has a help file that explains its ins & outs. `? PhyloFactor` especially contains a few gems, including surrogate data simulation and how to find Gaussian-shaped Hutchisonian niches.

I hope you enjoy the package! If you encounter any problems, or have some cool idea for phylofactorization that you’d like to see implemented, don’t be a stranger - drop me a line. You can contact me at

alex (dot) d (dot) washburne @ gmail.com

Cheers! -Alex
